# Toward Simulation of Fe(II) Low-Spin → High-Spin Photoswitching by Synergistic Spin-Vibronic Dynamics

**DOI:** 10.1021/acs.jctc.1c01184

**Published:** 2022-02-24

**Authors:** Mátyás Pápai

**Affiliations:** Wigner Research Centre for Physics, P.O. Box 49, H-1525 Budapest, Hungary

## Abstract

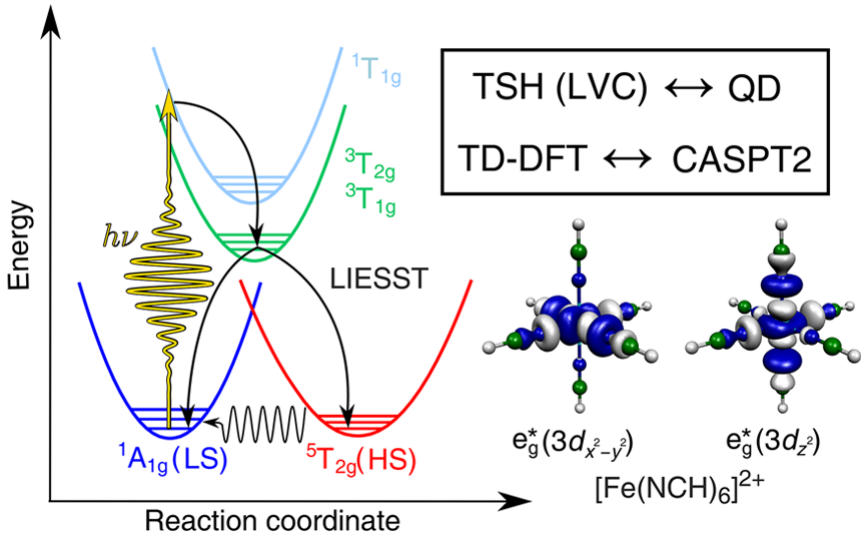

A new theoretical approach is presented and applied for the simulation of Fe(II) low-spin (LS, singlet, t_2g_^6^e_g_^0^) → high-spin (HS, quintet, t_2g_^4^e_g_^2^) photoswitching dynamics of the octahedral model complex [Fe(NCH)_6_]^2+^. The utilized synergistic methodology heavily exploits the strengths of complementary electronic structure and spin-vibronic dynamics methods. Specifically, we perform 3D quantum dynamics (QD) and full-dimensional trajectory surface hopping (TSH, in conjunction with a linear vibronic coupling model), with the modes for QD selected by TSH. We follow a hybrid approach which is based on the application of time-dependent density functional theory (TD-DFT) excited-state potential energy surfaces (PESs) and multiconfigurational second-order perturbation theory (CASPT2) spin–orbit couplings (SOCs). Our method delivers accurate singlet–triplet–quintet intersystem crossing (ISC) dynamics, as assessed by comparison to our recent high-level *ab initio* simulations and related time-resolved experimental data. Furthermore, we investigate the capability of our simulations to identify the location of ISCs. Finally, we assess the approximation of constant SOCs (calculated at the Franck–Condon geometry), whose validity has central importance for the combination of TD-DFT PESs and CASPT2 SOCs. This efficient methodology will have a key role in simulating LS → HS dynamics for more complicated cases, involving higher density of states and varying electronic character, as well as the analysis of ultrafast experiments.

## 1 Introduction

Low-spin (LS) → high-spin (HS) photoswitching, also known as light-induced excited spin-state trapping (LIESST),^1−4^ in transition metal (TM) complexes has been a very active scientific field in past decades. These investigations are motivated by both technological applications (e.g., molecular data storage^5,6^) and the fundamental importance of ultrafast excited-state processes.^7−9^ Time-resolved (pump–probe) experiments are powerful tools to resolve the excited-state dynamics leading to the LS → HS transition. However, the complexity of the recorded data often poses a very serious bottleneck, even if complementary experimental techniques, such as X-ray spectroscopy and scattering, are used. Theory, in particular dynamical techniques, thus has a key role in guiding the data analysis and designing ultrafast experiments.

Most of the LIESST-exhibiting TM complexes are based on Fe(II), in which case the singlet LS state (^1^A_1g_, t_2g_^6^e_g_^0^) is converted to a quintet HS state (^5^T_2g_, t_2g_^4^e_g_^2^). As a direct singlet–quintet intersystem crossing (ISC) involving a net Δ*S* = 2 change of the spin momentum is not possible (the spin–orbit coupling operator can only couple states with Δ*S* ≤ 1), Fe(II) LIESST can only proceed via intermediate triplet states. This means singlet–triplet–quintet dynamics, whose simulation poses immense challenges, as discussed below.

In the simpler case of singlet–triplet dynamics in TM complexes, i.e., when quintet states can be excluded, several computational dynamics works are known.^10^ In these studies, either quantum dynamics (QD) utilizing reduced-dimensionality model Hamiltonians^11−19^ or full-dimensional semiclassical trajectory surface hopping^20−24^ (TSH) is employed. The key to the success of these investigations is the description of excited states by an efficient linear-response (LR) time-dependent density functional theory (TD-DFT) methodology based on a singlet reference. Importantly, TD-DFT gives access to both potential energy surfaces (PESs) and singlet–triplet spin–orbit couplings (SOCs). However, quintet states cannot be computed by singlet-referenced LR TD-DFT, as it would require double excitations. Although this is possible using TD-DFT referenced to a quintet state, based on unrestricted DFT, the triplets and quintets obtained this way are incompatible for the calculation of triplet–quintet SOCs. An elegant solution to this problem is offered by multiconfigurational approaches, such as multiconfigurational second-order perturbation theory (CASPT2), by treating all spin states on the same footing. However, CASPT2 can be computationally rather expensive and is burdened by the often problematic selection of the active space.

**Figure 1:**
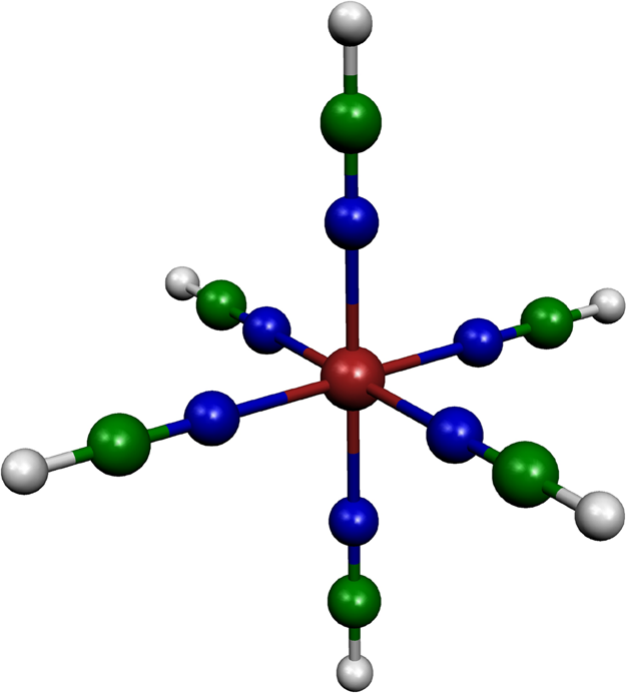
Molecular structure of the [Fe(NCH)_6_]^2+^ model complex.

Recently, we tackled the above electronic structure problem by utilizing the [Fe(NCH)_6_]^2+^ model^25^ (Figure 1) for LIESST in [Fe(ptz)_6_](BF_4_)_2_ (ptz = 1-propyltetrazole), which, in fact, is the first complex on which the LIESST mechanism was investigated.^3^ The application of the [Fe(NCH)_6_]^2+^ model allowed computation of CASPT2 PESs and SOCs, which we used to perform QD simulations with modes selected by full-dimensional TSH employing on-the-fly singlet–triplet TD-DFT potentials. A key to the success of this approach is the fact that solely metal-centered (MC, corresponding to d → d transitions) states are involved in LIESST in [Fe(ptz)_6_](BF_4_)_2_; thus, truncation of the ligands while keeping the same Fe^II^N_6_ core is justified. However, this model will certainly break down in several cases, such as the one of excitation into metal-to-ligand charge transfer (MLCT) states, exemplified by the famous prototypical [Fe(bipy)_3_]^2+^ (bipy = 2,2′-bipyridine) complex.^26−29^ CASPT2 calculations for larger molecules and/or higher density of states in most cases are not feasible, which thus hinders the simulation of excited-state dynamics. In this work, we explore the capability of a new synergistic approach for the [Fe(NCH)_6_]^2+^ LIESST model, in terms of both electronic structure (CASPT2/TD-DFT) and nuclear dynamics (QD/TSH). Our ultimate goal is to develop and apply an efficient methodology for the accurate simulation of LS → HS photoswitching dynamics.

## 2 Computational Methods

In this work we exploit the complementary character of both electronic structure and dynamical methods. We develop and employ a hybrid approach based on TD-DFT PESs and CASPT2 SOCs, both for full-dimensional (full-D) TSH and for QD, with modes selected by full-D TSH.

### 2.1 TSH Simulations

In our previous work on [Fe(NCH)_6_]^2+^,^25^ we used full-dimensional TSH with on-the-fly singlet–triplet TD-DFT potentials to select modes for QD. Here our aim is to include quintet states in the full-D TSH simulations. We use our CASPT2 SOCs from ref (25) (singlet–triplet, triplet–triplet, triplet–quintet, quintet–quintet), computed at the Franck–Condon (FC) geometry. However, these SOCs are not compatible with on-the-fly TSH based on the adiabatic basis, in which case the electronic character may vary as a function of the nuclear geometry. We overcome this obstacle by utilizing a diabatic linear vibronic coupling (LVC) model^24,30,31^ to calculate the potentials, which maintains the electronic character of the states. Apart from solving this SOC problem, the LVC approach is computationally very efficient.

The LVC-TSH methodology is described in detail in refs (24) and (31); here we only briefly summarize the key points. As the LVC model is based on normal modes, while TSH is carried out in Cartesian coordinates, first, the nuclear geometry is transformed to dimensionless mass-frequency weighted normal coordinates:
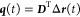
1where ***q***(*t*) and ***r***(*t*) are the time-dependent nuclear geometries (i.e., the trajectories) given in dimensionless normal and Cartesian coordinates, respectively, and ***D*** is the transformation matrix, based on the ground-state normal modes of the molecule. The diabatic potential energy matrix is then written as
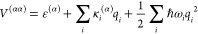
2
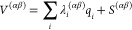
3

Here ℏ is the reduced Planck constant, ω_*i*_ is the vibrational frequency of mode *i*, ε^(α)^ is the vertical excitation energy from the ground state to state α at *q*_*i*_ = 0 for all *i* (i.e., at the FC geometry), κ^(α)^ and λ^(*αβ*)^ are the on-diagonal (intrastate) and off-diagonal (interstate) coupling constants, and *S*^(*αβ*)^ are the SOC matrix elements. κ^(α)^ and λ^(*αβ*)^ represent the nuclear gradients and nonadibatic couplings, respectively. Their values are determined by numerical differentiation, in the case of λ^(*αβ*)^, utilizing wave function overlaps,^32,33^ separately for spin-free singlet, triplet, and quintet states. The potential energy matrix ***V*** for the given nuclear geometry is then diagonalized to yield the diagonal basis used in the TSH simulations.

Our LVC model is based on DFT/TD-DFT employing the hybrid B3LYP* exchange–correlation functional^34,35^ and the TZVP basis set for all atoms. The B3LYP* functional was chosen on the basis of its known accuracy for Fe complexes.^36−39^ Two-electron integrals were approximated by the resolution of identity (RI-J) and chain of spheres (COSX) methods.^40^ For TD-DFT, we employ the Tamm–Dancoff approximation (TDA).^41^ Three singlet (^1^T_1g_) and six triplet (^3^T_1g_, ^3^T_2g_) excited states were computed using singlet-referenced (i.e., the ground state ^1^GS) TD-DFT, and three quintet (^5^T_2g_) states were computed using unrestricted quintet DFT and TD-DFT with two states on top of it. All DFT/TD-DFT calculations were carried out with the ORCA4.2 program package.^100,101^ For the determination of LVC parameters, we carried out these electronic structure calculations at the FC geometry as well as distorted along the modes using two-sided displacements at Δ*q*_*i*_ = ±0.5. The numerical values of the LVC parameters are given in the Supporting Information (Tables S2 and S6–S8 and the list of parameters below). We note that the Δ*q*_*i*_ = ±0.05 value utilized by previous works^23,31^ led to unrealistically large λ^(*αβ*)^ values for certain low-frequency modes, along which the PESs are rather flat. Finally, we tested whether artificial lowering of triplet states, reported in a previous LVC-TSH work,^23^ occurs for [Fe(NCH)_6_]^2+^. Using 500 geometries sampled from a ground-state Wigner distribution, we did not observe any negative (i.e., below the ground-state minimum) triplet energies, in fact, not even below 1 eV.

The TSH simulations were carried out with the SHARC2.1 code.^42^ The utilized TSH approach is based on Tully’s fewest switches,^43^ a three-step propagator technique (with transformations between the adiabatic/spin-diabatic and diagonal representations), and local diabatization.^44,45^ Time steps of 0.5 and 0.005 fs were used for the nuclear and electronic propagation, respectively. The gradient of all states calculated in the adiabatic/spin-diabatic representation was used for the transformation into the diagonal basis; correction by nonadiabatic couplings was applied in the gradient transformation. The energy-difference based correction scheme of Granucci et al.^46^ was used for decoherence correction with a decoherence parameter of 0.1 au. 500 initial conditions were sampled from a ground-state Wigner distribution, which were then used to run 500 trajectories for 1 ps, starting from the highest-energy adiabatic singlet state; this technique corresponds to excitation into the ^1^T_1g_ manifold. The single trajectory analyzed in section 3.3 was initiated from the FC geometry, with zero velocities, i.e., without any zero-point energy.

For the normal mode analysis presented in section 3.1, we use mass-frequency weighted normal coordinates *q*_*i,j*_ to characterize the activity of each mode *i* by the standard deviation calculated as
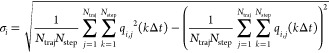
4where *j* and *k* run over all trajectories and time steps with upper bounds of *N*_traj_ = 500 and *N*_step_ = 2001, respectively.

### 2.2 QD Simulations

In ref (25), we carried out QD simulations utilizing a diabatic spin-vibronic model based on CASPT2 potentials along three selected modes. In this work, we assess the performance of QD based on TD-DFT (B3LYP*) PESs and CASPT2 SOCs; importantly, this hybrid methodology represents a significant step forward in terms of computation efficiency.

The spin-vibronic Hamiltonian used in this work is written as
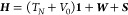
5with the kinetic energy operator
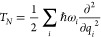
6and the zero-order potential
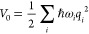
7for the ground-state harmonic oscillator. In eq 5, **1** is a unit matrix with a dimension equal to the number of electronic states included in the model, ***W*** is the potential energy matrix, and ***S*** is the SOC matrix. The elements of ***S***, identical to *S*^(*αβ*)^ in eq 3, are the CASPT2 SOCs taken from ref (25). For ***W***, we follow two different approaches: (i) LVC form with parameters taken for selected modes (full-dimensional QD simulations are not feasible due to very steep exponential scaling) from the model developed for the TSH simulations and (ii) extension the of LVC model with second-order on-diagonal terms γ_*i*_^(α)^ (expressing differences between excited and ground-state vibrational frequencies):
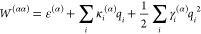
8
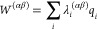
9with parameters determined by an energy-based method, known as diabatization by ansatz,^47^ in which the expansion coefficients are optimized such that the adiabatic potentials, obtained by diagonalization of ***W***, are in best agreement with adiabatic PESs calculated by quantum chemistry, in the present case, TD-DFT. We term this approach VCHAM, as it is used to construct a vibronic-coupling Hamiltonian. We used the same diabatization technique in ref (25) for the CASPT2 PESs of [Fe(NCH)_6_]^2+^. The numerical values of the parameters and fits to adiabatic PESs are given in the Supporting Information.

The DFT/TD-DFT calculations were carried out with the same setup as the one applied for the TSH simulations, described in section 2.1. The QD simulations were performed with the Heidelberg MCTDH8.4 code.^48^ The time-dependent Schrödinger equation was solved based on a wave function ansatz given by a multiconfigurational series of time-dependent Hartree (MCTDH) products of single particle functions (SPFs). The time-dependent SPFs are further expanded in a time-independent basis, in the present case, harmonic oscillator discrete variable representation (DVR). The QD simulations were performed using dimensionless mass-frequency scaled normal coordinates. The initial wavepacket (WP) is built of 1D harmonic oscillator eigenfunctions (with zero momentum). The ground-state WP is projected (impulsive excitation) onto a single component of the ^1^T_1g_ PES (α = 4). In Table 1, the primitive (*N*_*i*_) and SPF (*n*_*i*_^(α)^) basis sets are given, which ensured convergence for the 1 ps duration of the simulations.

**Table 1:** Primitive (*N*_*i*_) and SPF (*n*_*i*_^(α)^) Basis Set Sizes Used in the QD Simulations^a^

mode	*N*_*i*_	*n*_*i*_^(α)^
ν_13_	81	35, 35, 35, 35, 30, 30, 30, 30, 30, 30, 30, 30, 30, 30, 30, 30, 30, 30, 30, 30, 30, 30, 25, 25, 25, 25, 30, 25, 25, 25, 25, 25, 25, 30, 25
ν_14_	81	35, 35, 35, 35, 25, 30, 30, 30, 30, 30, 30, 30, 30, 30, 30, 30, 30, 30, 30, 30, 30, 30, 25, 25, 25, 25, 30, 25, 30, 25, 25, 25, 25, 25, 25
ν_15_	301	35, 35, 35, 35, 25, 25, 25, 25, 25, 25, 25, 25, 25, 25, 25, 25, 25, 25, 25, 25, 25, 25, 25, 25, 25, 25, 25, 25, 25, 25, 25, 25, 25, 25, 25

a The α electronic state index takes the following values: 1, ^1^GS; 2–4, ^1^T_1g_; 5–13, ^3^T_1g_; 14–22, ^3^T_2g_; 23–37, ^5^T_2g_.

### 2.3 Calculation of Spin–Orbit Couplings

In order to analyze the dependence of SOCs on the nuclear geometry, we carried out calculations based on multiconfigurational self-consistent field (CASSCF) and CASPT2 along the most important nuclear coordinates. The calculation of SOCs is based on the *ab initio* data presented in ref (25). We employ the Douglas–Kroll–Hess Hamiltonian (DKH)^49,50^ and an approach based on a one-electron effective mean-field SOC Hamiltonian and spin–orbit state interaction (SO-SI).^51,52^ CASSCF calculations were performed using a 14e,12o active space that includes the 5–5 Fe 3d and Fe 4d orbitals, two σ_Fe–N_ bonding orbitals, and a correlating pair of Fe 3s/Fe 4s orbitals. ANO-RCC basis sets^53−55^ were utilized with the following contractions: 7s6p5d4f3g2h for Fe, 4s3p2d1f for N, 4s3p1d for C, and 2s1p for H atoms. In the CASPT2 computations, we apply a 0.2 and 0.25 au imaginary level shift and IPEA shift,^56^ respectively. All CASSCF/CASPT2 computations were carried with the OpenMolcas20.10 quantum chemistry package.^57,58^ Further details of the CASSCF/CASPT2 methodology are given in ref (25). In section 3.4, we analyze absolute values of SOCs, as either the real part or the imaginary part (or both) cancels (cancel).

## 3 Results and Discussion

### 3.1 Mode Selection

As full-dimensional QD simulations for [Fe(NCH)_6_]^2+^ are not feasible, we here select the most important modes. For this purpose, we analyze the nuclear motion extracted from our full-dimensional TSH trajectories, including all relevant singlet, triplet, and quintet states.

**Figure 2:**
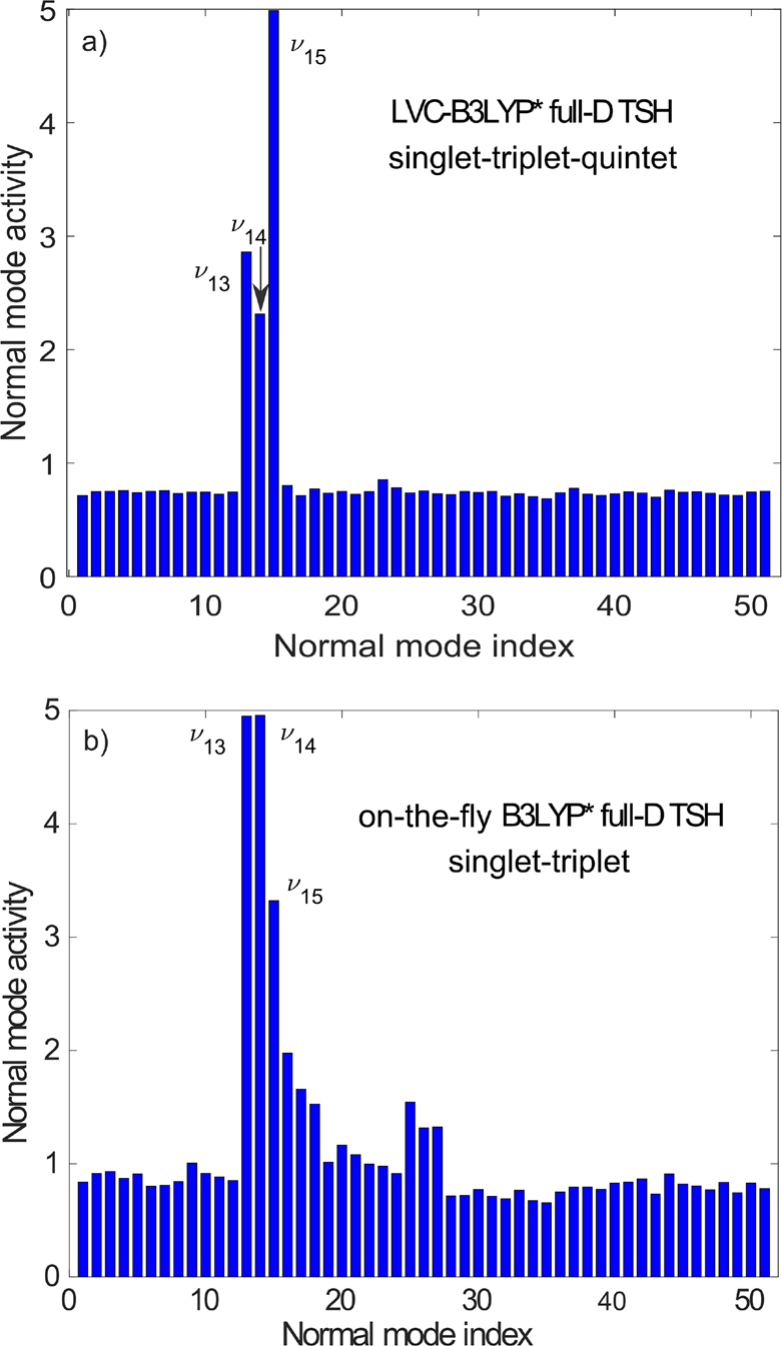
Dynamical normal mode activity, calculated as the standard deviation expressed in eq 4, for the full-dimensional TSH simulations employing (a) LVC singlet–triplet–quintet and (b) on-the-fly singlet–triplet potentials (from ref (25)). In both cases, the description of electronic structure is based on DFT/TD-DFT (B3LYP*). Character of the three dominant modes of Fe–N stretching: antisymmetric for ν_13_ and ν_14_ and totally symmetric (breathing) for ν_15_.

**Figure 3:**
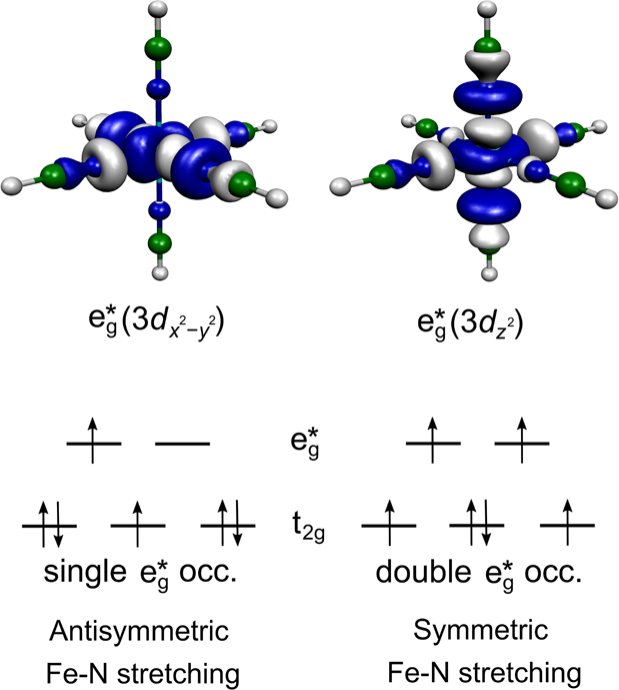
(top) Antibonding e_g_^*^ orbitals of [Fe(NCH)_6_]^2+^. (bottom) Examples of electronic configurations corresponding to single and double e_g_^*^ occupations. A single e_g_^*^ occupation occurs for the ^1^T_1g_, ^3^T_1g_, and ^3^T_2g_ states, here shown for a triplet MC configuration. Double e_g_^*^ occupation occurs for quintet states (^5^T_2g_, HS). The character of triggered Fe–N vibrations upon electronic d → d excitation is also shown.

Figure 2a shows the dynamical normal mode activity for the TSH simulation, clearly identifying the dominance of three modes, ν_13_, ν_14_, and ν_15_. All three modes have Fe–N stretching character. ν_13_ and ν_14_ are a degenerate pair (e_g_ within *O*_*h*_ symmetry) with antisymmetric Fe–N stretching character, while ν_15_ is a totally symmetric (a_g_) Fe–N stretching (breathing) mode. The dominance of these three Fe–N stretching modes is in line with the fact that solely MC excited states are involved in the dynamics. Namely, a single occupation of an antibonding e_g_^*^ orbital (singlet and triplet states, see Figure 3) activates an antisymmetric Fe–N stretching mode, while in the case of double e_g_^*^ occupation (quintet state) an Fe–N breathing vibration is triggered. Importantly, the same three modes were identified for full-dimensional TSH based on on-the-fly singlet–triplet TD-DFT potentials in our previous study,^25^ i.e., with no quintet states but full quantum chemical PESs without any restrictions such as its harmonicity; the mode activity result for this method is displayed in Figure 2b. This consistency supports the selection of three Fe–N stretching modes. We note that the increased activity of breathing mode ν_15_ for the LVC-TSH simulation (Figure 2a) is in agreement with the inclusion of quintet states (double e_g_^*^ occupation, which activates the totally symmetric breathing mode).

### 3.2 Synergistic Spin-Vibronic LIESST Dynamics

The LIESST dynamics of [Fe(ptz)_6_]^2+^ and our model, [Fe(NCH)_6_]^2+^, involves solely MC excited states: the singlet ^1^T_1g_, the triplets ^3^T_1g_ and ^3^T_2g_, and the quintet ^5^T_2g_, with “T” denoting triple degeneracy at the FC geometry (*O*_*h*_ symmetry).

**Figure 4:**
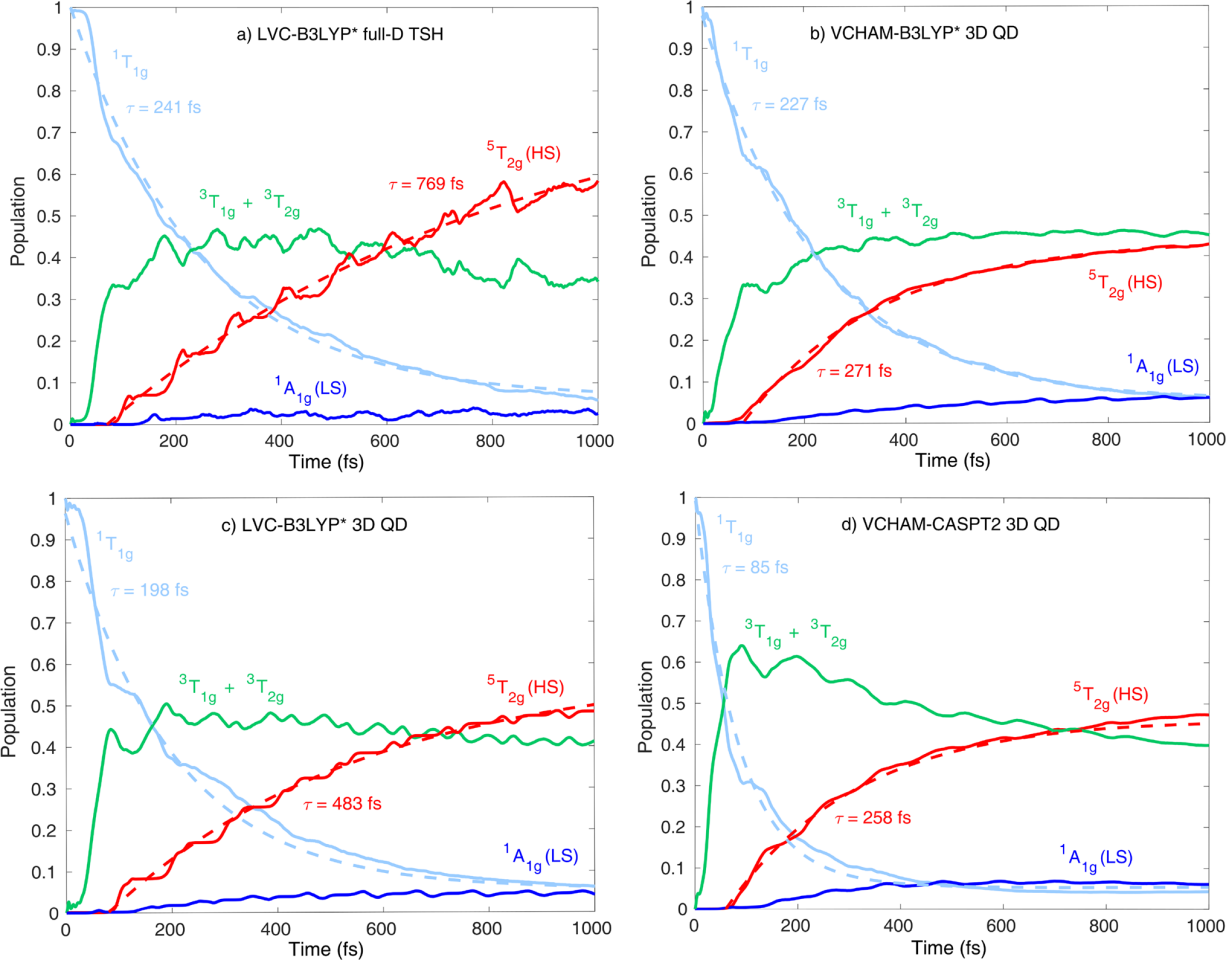
Simulated population dynamics upon ^1^T_1g_ photoexcitation. The four panels show the results for different approaches: (a) full-dimensional TSH on LVC-B3LYP* potentials and 3D-QD on (b) VCHAM-B3LYP*, (c) LVC-B3LYP*, and (d) VCHAM-CASPT2 PESs. Note that the TSH and QD populations are made consistent by summing up the excited-state populations for the same spin multiplicity (singlet, triplet, quintet). The dashed lines represent exponential fits with the functions exp(−*t*/τ) and 1 – exp(−*t*/τ) for ^1^T_1g_ and ^5^T_2g_, respectively.

In Figure 4, we compare the excited-state population dynamics of [Fe(NCH)_6_]^2+^, promoted to the ^1^T_1g_ states, for full-dimensional TSH (LVC-B3LYP*) and 3D-QD with the selected modes ν_13_, ν_14_, and ν_15_ (VCHAM-B3LYP*, LVC-B3LYP*, CASPT2-VCHAM). Here, in order to avoid problems that may arise from diabatization of TSH adiabatic/spin-diabatic populations, we added up those excited-state populations that correspond to the same spin multiplicity (singlet, triplet, quintet). The overall agreement between the dynamics simulated by the four methods is good, which is a very positive result, especially in terms of differences in the theoretical approaches, both nuclear dynamics (full-D TSH versus 3D-QD) and electronic structure (DFT/TD-DFT versus CASPT2, LVC versus VCHAM). In all four cases, the initially excited ^1^T_1g_ states (light blue in Figure 4) decay via ISC to the triplet states with an exponential time constant ranging from 85 fs (CASPT2 3D-QD, Figure 4d) to 241 fs (LVC-B3LYP* full-D TSH, Figure 4a); thus a good agreement is obtained. All simulated singlet–triplet ISC rates are in line with the <150 fs lifetime from the time-resolved experiment^59^ on [Fe(ptz)_6_](BF_4_)_2_, doped into a Zn matrix; the CASPT2 QD value of 85 fs even reaches a quantitative agreement with the experimental value. In all cases, the rise of the quintet ^5^T_2g_ population is slightly delayed with respect to the one of the triplets, in line with the fact that the singlet–quintet transition has to proceed via the triplet states. For all three QD methods (Figure 4b–d) the rise of the quintet population (red in Figure 4) significantly slows down after 500–600 fs, leading to exponential rise constants below 500 fs. In contrast, in the case of the TSH simulation (Figure 4a), the quintet population rise is more continuous, resulting in a rise time constant of 769 fs, in slightly better agreement with the 1.2 ps time constant for triplet–quintet ISC. We interpret this difference between TSH and QD, for >500 fs, as the indirect effect of the bath of vibrational modes, which is absent in our QD models and, as observed in Figure 4a, can lead to a more efficient transition into the quintet HS state. The participation of triplet states is evident in all four cases (green curves in Figure 4). However, while the triplet population curve for the CASPT2 QD simulation exhibits a clear decay (Figure 4d), this decaying component is less pronounced for the other three methods based on B3LYP*. In fact, for VCHAM-B3LYP* QD (Figure 4b), no decay is observed. Finally, the weak population of the ground state ^1^A_1g_ (dark blue in Figure 4) appears in all cases as a minor component.

### 3.3 TSH LIESST Dynamics: Locating ISCs

**Figure 5:**
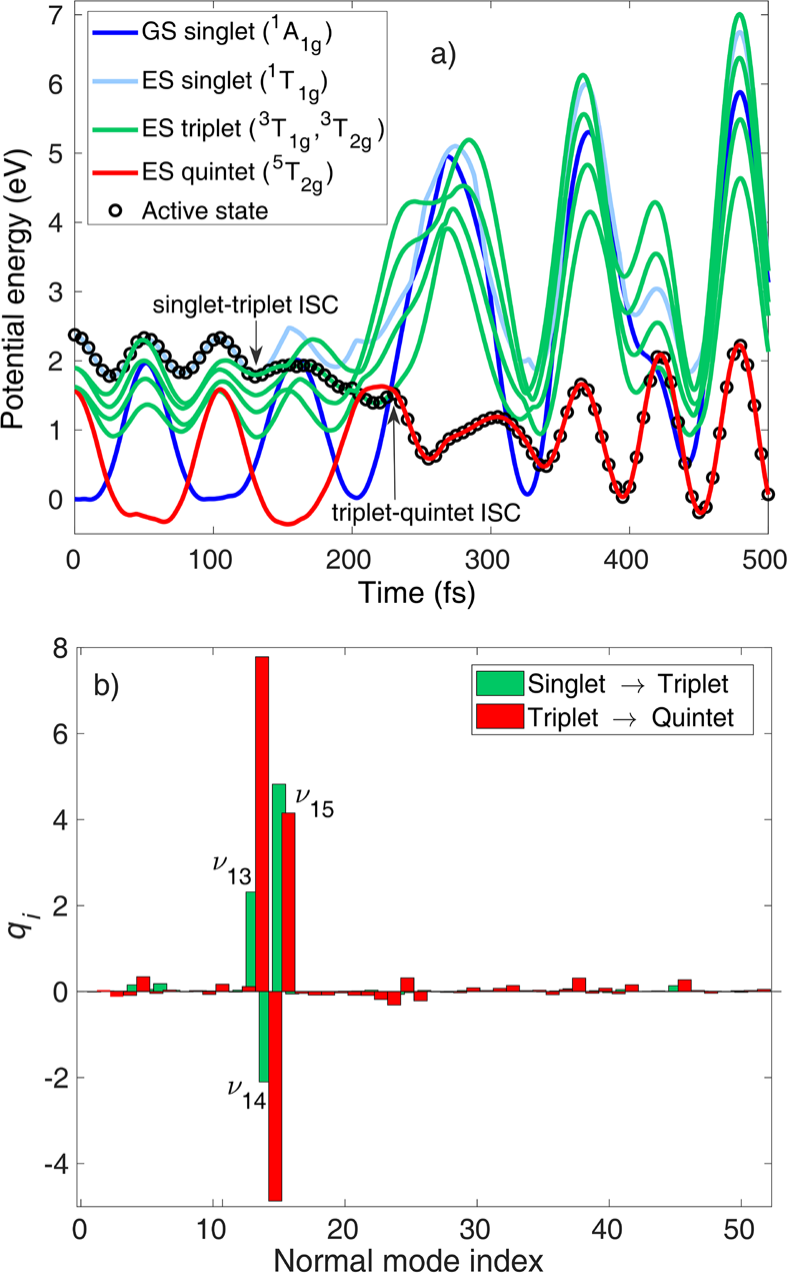
(a) Adiabatic/spin-diabatic potential energies along a trajectory initiated from the S_3_ (^1^T_1g_) state at the FC geometry with zero velocities. The active (classically populated) state is marked by circles. For clear visibility, the energies of the two upper singlet (S_2_, S_3_) and quintet (Q_2_, Q_3_) excited states as well as the T_2_ and T_5_ triplet states are not shown. (b) ISC (hopping) geometries marked in (a), given in dimensionless mass-frequency weighted normal coordinates: green, singlet–triplet ISC; red, triplet–quintet ISC.

In addition to the selection of modes, TSH offers powerful tools to interpret excited-state mechanisms, e.g., identification of the location of singlet–triplet and triplet–quintet ISCs. Figure 5a shows the potential energies along a single trajectory (FC geometry, zero velocities). We note that this simple case is not meant for quantitative analysis but is meant to qualitatively visualize the ISC dynamics. As seen in Figure 5, the initially excited singlet state becomes degenerate with the highest-lying triplet within 40 fs, driven by the ^1^T_1g_ nuclear gradient around the FC geometry. Afterward, a singlet–triplet ISC occurs at 130 fs. The geometry at which the transition occurs is given in dimensionless normal coordinates in Figure 5b (green). In agreement with our previous findings, this demonstrates the clear dominance of the three Fe–N stretching modes ν_13_–ν_15_. After propagating on the triplet PES, the trajectory reaches a triplet–quintet intersection at 205 fs, close to which the triplet ^3^T_1g_ and ^3^T_2g_ potentials also cross, and a triplet–quintet crossing is observed at 230 fs. The triplet–quintet ISC occurs at 230 fs at the geometry in normal mode coordinates displayed in Figure 5b (red). This triplet–quintet ISC geometry is highly distorted along the Fe–N stretching coordinates, with the largest contribution from ν_13_ and negligible displacement along the other modes. Thereafter, the trajectory propagates on the quintet surface; the nuclear motion here is dominated by Fe–N breathing (ν_15_), which is activated by the double occupation of e_g_^*^ orbitals, and is reflected in Figure 5a between 230 and 500 fs by the large-amplitude oscillations in the singlet and triplet energies. Further triplet–quintet switches only occur after 500 fs (quintet–triplet and triplet–quintet transitions at ca. 650 and 830 fs, respectively).

**Figure 6:**
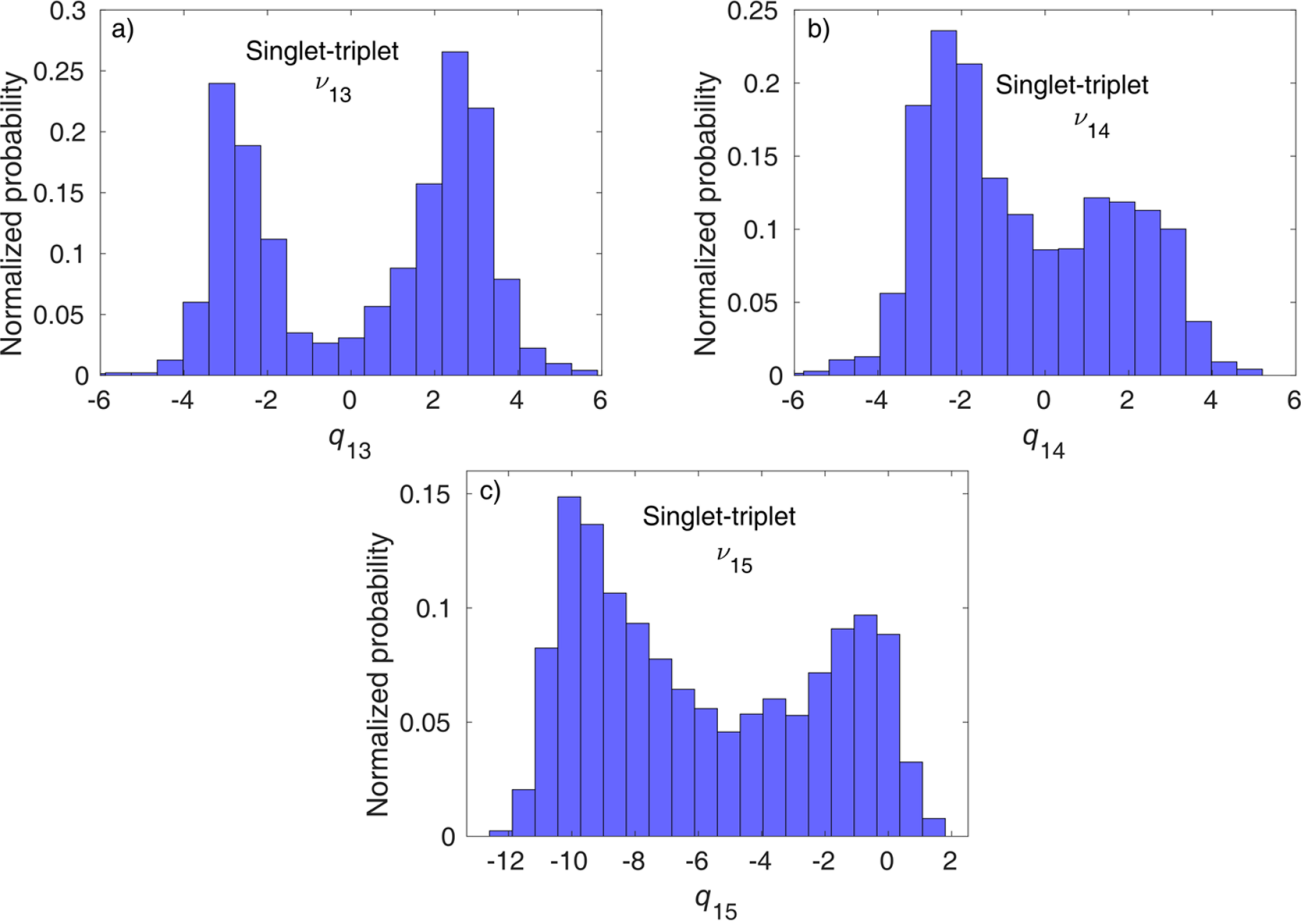
Distribution of singlet–triplet hopping geometries, by projection to dimensionless mass-frequency weighted normal mode coordinates. The three panels show the distributions for (a) ν_13_, (b) ν_14_, and (c) ν_15_. The area of the distributions (histograms) is normalized to unity.

We now switch from the analysis of a single trajectory to the ensemble of all trajectories, from which we identify the location of ISCs by the distribution of singlet–triplet and triplet–quintet hopping geometries. In agreement with our findings, the nuclear distortions from the FC geometry for these ISC structures are clearly dominated by the three Fe–N stretching modes, ν_13_, ν_14_, and ν_15_. In Figure 6, we present the distributions of the singlet–triplet hopping geometries for the three modes. It is clear from Figure 6 that the distributions are most structured and localized along ν_13_, which is thus identified as the decisive mode for singlet–triplet ISC. In contrast, the distributions along the two other modes, ν_14_ and, especially, ν_15_, shown in parts b and c, respectively, of Figure 6, are broader and less structured, which is explained by weaker vibronic effects. Therefore, ν_13_ is assigned as the principal mode for singlet–triplet ISC.

**Figure 7:**
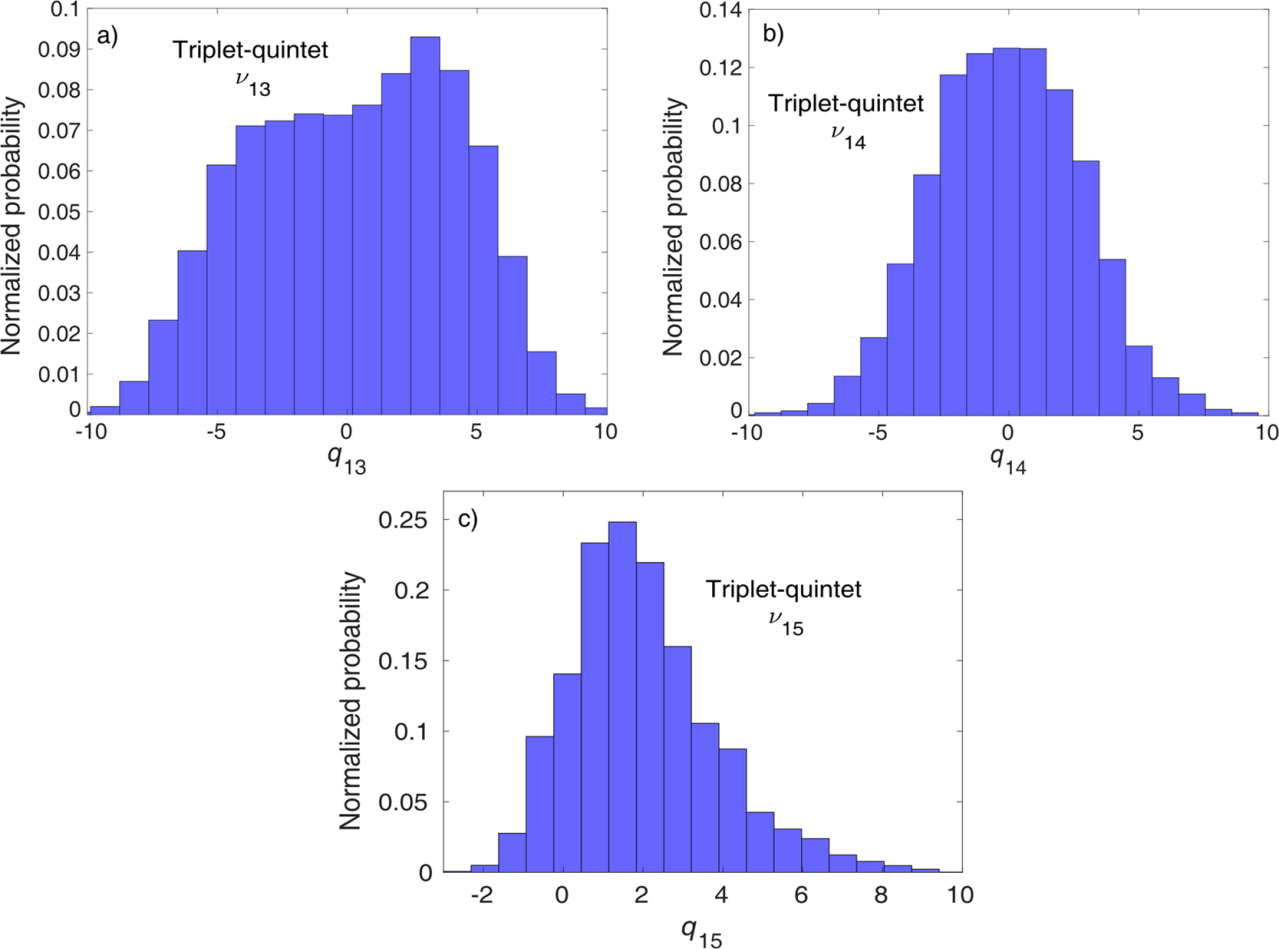
Distribution of triplet–quintet hopping geometries, by projection to dimensionless mass-frequency weighted normal mode coordinates. The three panels show the distributions for (a) ν_13_, (b) ν_14_, and (c) ν_15_. The area of the distributions (histograms) is normalized to unity.

Figure 7 shows the distribution of triplet–quintet switching geometries. As opposed to the singlet–triplet transitions, the distribution for ν_13_ is very broad (Figure 7a); for the other antisymmetric stretching mode, ν_14_, the distribution is narrower but still broad (Figure 7b). The situation is quite different for the breathing mode, ν_15_, along which the distribution is the narrowest (Figure 7c), and it is thus identified as the dominant mode for triplet–quintet transitions. The distribution along ν_15_ is centered around ca. *q*_15_ = 1, which is close to the triplet–quintet intersection along ν_15_ for ^3^T_1g_ (*q*_15_ ∼ 0) and slightly further for ^3^T_2g_ (*q*_15_ ∼ −1.5, see Figure S8). This result and the one obtained for the single trajectory, i.e., a ^3^T_1g_/^3^T_2g_ intersection is reached just before the triplet–quintet crossing, shown in Figure 5, indicate that the triplet–quintet transition occurs, at least partially via the ^3^T_1g_ state, and thus point toward the coupling of triplet–quintet ISC and triplet–triplet internal conversion (IC) pathways. We note that this ISC–IC competition can be affected by the electronic structure description: although the overall agreement of B3LYP* and CASPT2 PESs is rather good, the ^3^T_1g_–^3^T_2g_ triplet energy gap is nearly twice as large for CASPT2 (0.33 eV) as for B3LYP* (0.19 eV), altering the triplet IC dynamics (see Table S2 and Figure S9).

For further analysis, in the Supporting Information (section S5), we detail the TSH and QD vibrational dynamics along the dominant modes identified for singlet–triplet and triplet–quintet ISCs, ν_13_ and ν_15_, respectively. Briefly, we find that while ν_13_ is characterized by out-phase vibrations and broadening, coherent oscillations with a period of ca. 100 fs and slow coherenece decay dominate the breathing motion ν_15_.

### 3.4 Methodological Aspects

Importantly, from comparison with the CASPT2 dynamics and experimental time scales, we find that our hybrid approach, based on DFT/TD-DFT PESs (both LVC and VCHAM) and CASPT2 SOCs, performs reasonably well. This is a very promising result for simulating photoswitching dynamics in larger complexes with a computational bottleneck of a single CASPT2 calculation (at the FC geometry); compared to this, the computational effort of all corresponding DFT/TD-DFT calculations is almost negligible. In addition, the utilized synergistic TSH–QD methodology offers an internal convergence check for the reliability of the results. TSH, as it treats the nuclear motion classically, has the powerful capability to be carried out in full dimension; however, feedback from QD for the most important modes is important to assess the description of quantum effects. Conversely, TSH, in addition to selecting the modes for QD, which can only operate for TM complexes in reduced dimension, can validate the adequacy of the chosen modes by contrasting the TSH and QD dynamics. We propose the LVC model for this analysis, whose parameters can be efficiently calculated, even in full dimension, and ensures the same level of theory for TSH and QD. Finally, the validity of the LVC method can be verified by comparison to results obtained by VCHAM including the second-order term γ_*i*_^(α)^. In the present case for [Fe(NCH)_6_]^2+^, the agreement is rather good and the application of the LVC model is thus justified. This is also supported by the LVC-VCHAM PESs along the three Fe–N stretching modes, shown in Figures S1 and S2. Namely, the only significant difference with respect to the VCHAM PESs, relevant for the dynamics, is that the HS–LS energy gap for LVC along the breathing mode ν_15_ is lowered by ∼0.2–0.3 eV (note that athough the utilization of ground-state vibrational frequencies in the LVC model increases the gap, a lowering is yet observed, caused by larger quintet κ_15_^(α)^ values than for VCHAM, see Tables S3 and S8). However, as is clear from Figure 4, this variation in the PESs along ν_15_ does not lead to any qualitative differences in the simulated dynamics.

All results presented so far are based on spin–orbit couplings calculated at the FC geometry, not taking into account their dependence on the nuclear coordinates. This approach has been widely utilized in dynamical computational studies, albeit in most cases without any justification; rare exceptions include analysis of singlet–triplet SOCs for Cu^11^ and Re^60^ complexes. We here fill this gap for [Fe(NCH)_6_]^2+^ by assessing the variation of singlet–triplet and triplet–quintet SOCs along the antisymmetric Fe–N stretching mode ν_13_ and the breathing mode ν_15_.

**Figure 8:**
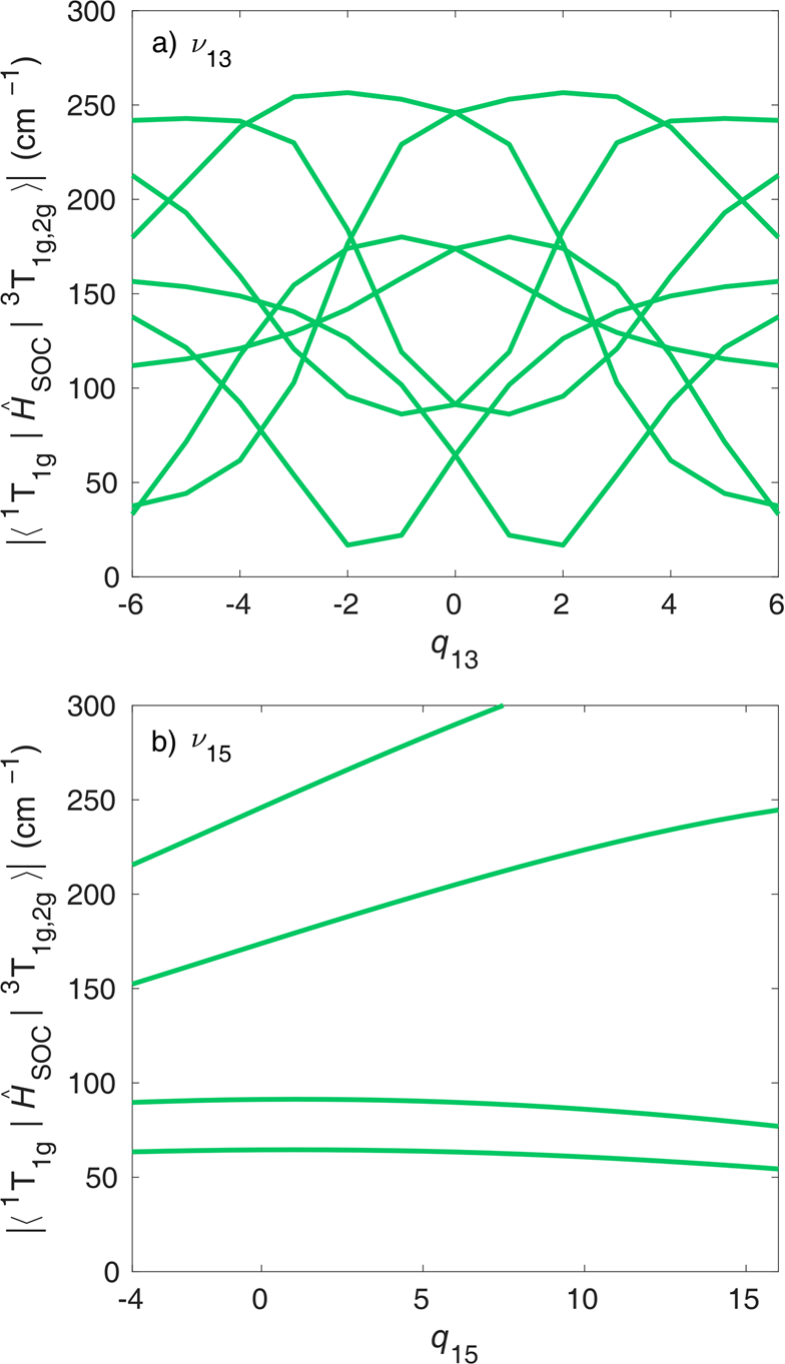
Absolute value of singlet–triplet CASPT2 spin–orbit couplings along (a) ν_13_ and (b) ν_15_.

**Figure 9:**
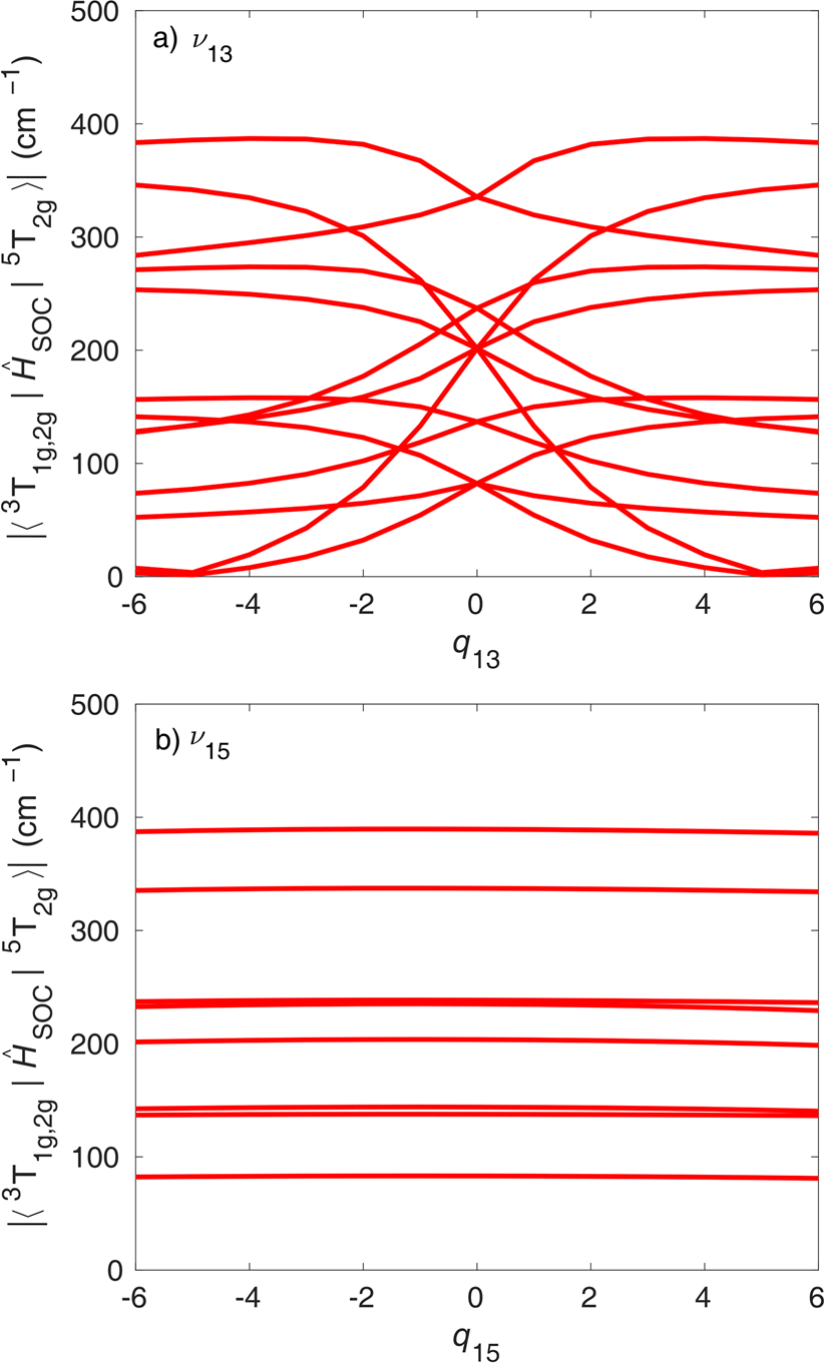
Absolute value of triplet–quintet CASPT2 spin–orbit couplings along (a) ν_13_ and (b) ν_15_. For clarity, not all triplet–quintet SOCs are shown.

Figure 8 shows the singlet–triplet SOCs along ν_13_ and ν_15_. First, all SOCs that are zero at the FC geometry also vanish at the displaced geometries (not shown in Figure 8); this is also true for the triplet–quintet SOCs. This result is an important finding, because ν_13_ breaks the octahedral symmetry, which could lift the symmetry rules, and thus the approach of FC-based SOCs would fail, but it is clearly not the case. Figure 8 shows that, in most cases, the geometry dependence of singlet–triplet SOCs is significant but still much smaller than the variation of energy gaps during the dynamics. Importantly, no very drastic changes are observed; e.g., an SOC element vanishes. For the totally symmetric mode ν_15_, the singlet–triplet SOCs exhibit nearly linear dependence. Figure 9 displays the triplet–quintet SOCs. In general, the triplet–quintet SOCs are larger than the triplet–quintet SOCs and, more importantly, less dependent on the nuclear geometry, which means that the approximation of FC SOCs is even better for triplet–quintet SOCs. Nevertheless, certain SOC elements along ν_13_ do go to zero, but note that (i) this occurs at relatively large distortions (*q* ∼ ±5), while the majority of triplet–quintet transitions along ν_13_ occur at smaller distortions (see Figure 7a), and (ii) this behavior is only observed for a very few out of all nonzero SOC elements. Finally, all triplet–quintet SOCs along ν_15_ exhibit a negligible dependence on the nuclear geometry.

## 4 Conclusion

In this work, we performed synergistic spin-vibronic dynamics simulations on the LIESST model [Fe(NCH)_6_]^2+^, solely including metal-centered excited states. The methodology is based on an efficient hybrid approach utilizing DFT/TD-DFT PESs/CASPT2 SOCs, and the selection of dominant modes by full-dimensional TSH. For TSH, we employ a wave-function-based singlet–triplet–quintet LVC method, while for QD we use both LVC and an energy-based diabatization scheme. In agreement with our recent study,^25^ we observe the clear dominance of the three Fe–N stretching modes, the antisymmetric modes ν_13_ and ν_14_ and the totally symmetric (breathing) mode ν_15_. Importantly, the dynamics simulated by our new hybrid approach, both with TSH and QD, show good overall agreement with our recent CASPT2 simulations^25^ as well as time-resolved experiments performed on the analogous [Fe(ptz)_6_](BF_4_)_2_ complex. Specifically, we identify a fast ∼200 fs singlet–triplet ISC and a slightly slower ∼300–800 fs triplet–quintet ISC. The consistency of TSH–QD dynamics, obtained by the hybrid TD-DFT/CASPT2 approach, serves as an internal feedback for the reliability of the results.

Next, we exploit the capability of our TSH methodology to determine the location of ISCs, based on the distribution of hopping geometries. We find that ISCs occur near the intersection of excited-state potentials, with the antisymmetric mode ν_13_ being dominant for singlet–triplet ISC and the breathing mode ν_15_ dominant for triplet–quintet ISC. This result is in agreement with the activation of Fe–N stretching coordinates caused by single (singlet and triplet excited states, ^1^T_1g_ and ^3^T_1g,2g_) and double (quintet states, ^5^T_2g_) occupation of antibonding e_g_^*^ orbitals. We also find indications of coupling of triplet IC and triplet–quintet ISC pathways.

Finally, we assess the dependence of singlet–triplet and triplet–quintet SOCs on the nuclear geometry, along the three Fe–N stretching modes, and thus the adequacy of the constant FC SOC model. Although several SOC elements do exhibit a significant geometry dependence, this is small relative to the variation of energy gaps during the dynamics. Importantly, departure from octahedral symmetry does not lead to appearance of SOCs that are zero at the FC geometry. The opposite effect, i.e., vanishing SOCs at distorted geometries, does occur, but very rarely and only at large nuclear distortions. We thus conclude that the constant SOC model is a reasonable approach for the dynamics of [Fe(NCH)_6_]^2+^, which is a key result for establishing our hybrid methodology (the combination of TD-DFT PESs and geometry-dependent CASPT2 SOCs would be problematic, from a fundamental point of view).

## Supporting Information Available

Tabulated normal modes, vibronic-coupling parameters and spin–orbit couplings, diabatic and adiabatic/spin-diabatic potential energy surfaces, comparison of VCHAM-B3LYP* and VCHAM-CASPT2 QD, description of vibrational dynamics by TSH and QD (PDF)

## Notes

DTU Computing Center, DTU Computing Center resources. 2021; https://doi.org/10.48714/DTU.HPC.0001.
