# Supplementary material for: Toward Simulation of Fe(II) Low-Spin → High-Spin Photoswitching by Synergistic Spin-Vibronic Dynamics
Source: J Chem Theory Comput. 2022 Feb 24;18(3):1329–39. doi: 10.1021/acs.jctc.1c01184 (PMC8908767; doi:10.1021/acs.jctc.1c01184)
Supplement: Supplementary file 1 — ct1c01184_si_001.pdf [file ct1c01184_si_001.pdf]

Supporting Information for "Towards Simulation of Fe(II) Low-Spin → High-Spin Photoswitching by Synergistic Spin-Vibronic Dynamics"

Mátyás Pápai*

Wigner Research Centre for Physics, P.O. Box 49, H-1525 Budapest, Hungary

Email: papai.matyas@wigner.hu

Contents

S1 Normal Modes **S2**

S2 Vibronic Coupling Parameters and Spin-Orbit Couplings **S3**

S3 Diabatic and Adiabatic/Spin-Diabatic Potentials **S22**

S4 Comparison of B3LYP* and CASPT2 QD **S31**

S5 Description of Vibrational Dynamics by TSH and QD **S31**

S1 Normal Modes

Table S1 reports the ground-state normal modes of $[Fe(NCH)_6]^{2+}$, calculated at the B3LYP*/TZVP level of theory:

Table S1: DFT-caculated (B3LYP*/TZVP) ground-state normal modes of $[Fe(NCH)_6]^{2+}$.

Mode	Symmetry (O_h)	Freq. (cm^{-1})	Character
ν_1	t_{2u}	87.1	Rocking, bending (Fe-N-C)
ν_2	t_{2u}	87.1	Rocking, bending (Fe-N-C)
ν_3	t_{2u}	87.1	Rocking, bending (Fe-N-C)
ν_4	t_{2g}	106.7	Rocking, bending (Fe-N-C)
ν_5	t_{2g}	106.7	Rocking, bending (Fe-N-C)
ν_6	t_{2g}	106.7	Rocking, bending (Fe-N-C)
ν_7	t_{1u}	125.0	Rocking, bending (Fe-N-C)
ν_8	t_{1u}	125.0	Rocking, bending (Fe-N-C)
ν_9	t_{1u}	125.0	Rocking, bending (Fe-N-C)
ν_{10}	t_{1g}	248.6	Rocking, bending (Fe-N-C)
ν_{11}	t_{1g}	248.6	Rocking, bending (Fe-N-C)
ν_{12}	t_{1g}	248.6	Rocking, bending (Fe-N-C)
**ν_{13}**	**e_g**	**299.1**	**Antisymmetric stretching (Fe-N)**
**ν_{14}**	**e_g**	**299.1**	**Antisymmetric stretching (Fe-N)**
**ν_{15}**	**a_{1g}**	**´318.4**	**Symmetric stretching (Fe-N)**
ν_{16}	t_{1u}	334.3	Fe off-center movement, bending (Fe-N-C)
ν_{17}	t_{1u}	334.3	Fe off-center movement, bending (Fe-N-C)
ν_{18}	t_{1u}	334.3	Fe off-center movement, bending (Fe-N-C)
ν_{19}	t_{2u}	361.1	Twisting (Fe-N-C, N-Fe-N)
ν_{20}	t_{2u}	361.1	Twisting (Fe-N-C, N-Fe-N)
ν_{21}	t_{2u}	361.1	Twisting (Fe-N-C, N-Fe-N)
ν_{22}	t_{2g}	376.0	Bending (Fe-N-C, N-Fe-N)
ν_{23}	t_{2g}	376.0	Bending (Fe-N-C, N-Fe-N)
ν_{24}	t_{2g}	376.0	Bending (Fe-N-C, N-Fe-N)
ν_{25}	t_{1u}	464.8	Bending (Fe-N-C, N-Fe-N)
ν_{26}	t_{1u}	464.8	Bending (Fe-N-C, N-Fe-N)
ν_{27}	t_{1u}	464.8	Bending (Fe-N-C, N-Fe-N)
ν_{28}	t_{1g}	786.0	Bending (N-C-H)
ν_{29}	t_{1g}	786.0	Bending (N-C-H)
ν_{30}	t_{1g}	786.0	Bending (N-C-H)
ν_{31}	t_{2u}	787.0	Bending (N-C-H)
ν_{32}	t_{2u}	787.0	Bending (N-C-H)
ν_{33}	t_{2u}	787.0	Bending (N-C-H)
ν_{34}	t_{1u}	787.7	Bending (N-C-H)
ν_{35}	t_{1u}	787.7	Bending (N-C-H)
ν_{36}	t_{1u}	787.7	Bending (N-C-H)

ν_{37}	t_{2g}	788.9	Bending (N-C-H)
ν_{38}	t_{2g}	788.9	Bending (N-C-H)
ν_{39}	t_{2g}	788.9	Bending (N-C-H)
ν_{40}	e_g	2238.4	Antisymmetric stretching (N-C)
ν_{41}	e_g	2238.4	Antisymmetric stretching (N-C)
ν_{42}	t_{1u}	2239.1	Antisymmetric stretching (N-C)
ν_{43}	t_{1u}	2239.1	Antisymmetric stretching (N-C)
ν_{44}	t_{1u}	2239.1	Antisymmetric stretching (N-C)
ν_{45}	a_{1g}	2249.8	Symmetric stretching (N-C)
ν_{46}	e_g	3373.1	Antisymmetric stretching (C-H)
ν_{47}	e_g	3373.1	Antisymmetric stretching (C-H)
ν_{48}	t_{1u}	3373.2	Antisymmetric stretching (C-H)
ν_{49}	t_{1u}	3373.2	Antisymmetric stretching (C-H)
ν_{50}	t_{1u}	3373.2	Antisymmetric stretching (C-H)
ν_{51}	a_{1g}	3378.7	Symmetric stretching (C-H)

S2 Vibronic Coupling Parameters and Spin-Orbit Couplings

All below parameters were obtained by using DFT/TD-DFT (B3LYP*/TZVP) quantum chemistry for the following diabatic Hamiltonians: i) VCHAM-B3LYP* and ii) LVC-B3LYP*. For i), we introduced the upper S_4−S_6 singlet states for VCHAM fitting along the two antisymmetric modes ν_{13} and ν_{14}; this is required because the $^1T_{1g}$ and $^1T_{2g}$ manifolds interact along ν_{13} and ν_{14}. The dynamics simulations were performed with the $^1T_{1g}$ singlet states only, as the $^1T_{2g}$ states are energetically inaccessible. The utilized spin-orbit coupling (SOC) matrix[1] is given in Tables S9−S14 and the *ab initio* data for the calculation of SOCs along the ν_{13} and ν_{15} Fe-N stretching modes are given in ref. 1.

Table S2: Zeroth-order coefficients $\varepsilon^{(\alpha)}$, given in eV. The tabulated $\varepsilon^{(\alpha)}$ values are the B3LYP*-calculated (DFT/TD-DFT) vertical excitation energies at the Franck-Condon (FC) geometry. Both FC adiabatic and diabatic state labels, the latter in parantheses, are given. Note that the S_4, S_5, S_6 ($^1T_{2g}$) states were not used in the QD simulations but are reported here for completeness. These B3LYP* $\varepsilon^{(\alpha)}$ values were used in the simulations both with VCHAM-B3LYP* and LVC-B3LYP*. For comparison, the CASPT2 values from ref. 1 are also shown.

State	$\varepsilon^{(\alpha)}$ – B3LYP*	$\varepsilon^{(\alpha)}$ – CASPT2
S_0 (^{1}GS)	0.000	0.000
S_1 ($^1T_{1g}$)	2.368	2.266
S_2 ($^1T_{1g}$)	2.368	2.266
S_3 ($^1T_{1g}$)	2.368	2.266
S_4 ($^1T_{2g}$)	3.384	3.251
S_5 ($^1T_{2g}$)	3.384	3.251
S_6 ($^1T_{2g}$)	3.384	3.251
T_1 ($^3T_{1g}$)	1.624	1.280
T_2 ($^3T_{1g}$)	1.624	1.280
T_3 ($^3T_{1g}$)	1.624	1.280
T_4 ($^3T_{2g}$)	1.812	1.812
T_5 ($^3T_{2g}$)	1.812	1.876
T_6 ($^3T_{2g}$)	1.812	1.876
Q_1 ($^5T_{2g}$)	1.577	1.333
Q_2 ($^5T_{2g}$)	1.647	1.333
Q_3 ($^5T_{2g}$)	1.647	1.333

Table S3: First-order diagonal coefficients $\kappa_i^{(\alpha)}$ for VCHAM-B3LYP*, given in eV. Both FC adiabatic and diabatic state labels, the latter in parantheses, are given. Note that the S_4–S_6 ($^1T_{2g}$) states were not used in the QD simulations but are reported here for completeness.

State	$\kappa_{13}^{(\alpha)}$	$\kappa_{14}^{(\alpha)}$	$\kappa_{15}^{(\alpha)}$
S_0 (^{1}GS)	0.023	0.031	0.010
S_1 ($^1T_{1g}$)	0.080	0.103	0.145
S_2 ($^1T_{1g}$)	0.023	−0.032	0.145
S_3 ($^1T_{1g}$)	−0.040	0.015	0.145
S_4 ($^1T_{2g}$)	−0.030	−0.040	N/A
S_5 ($^1T_{2g}$)	0.019	0.004	N/A
S_6 ($^1T_{2g}$)	0.073	0.105	N/A
T_1 ($^3T_{1g}$)	0.055	0.070	0.154
T_2 ($^3T_{1g}$)	0.069	0.077	0.154
T_3 ($^3T_{1g}$)	−0.046	−0.056	0.154
T_4 ($^3T_{2g}$)	−0.010	−0.011	0.146
T_5 ($^3T_{2g}$)	−0.028	−0.029	0.146
T_6 ($^3T_{2g}$)	0.081	0.096	0.146
Q_1 ($^5T_{2g}$)	0.013	0.031	0.322
Q_2 ($^5T_{2g}$)	0.020	0.011	0.331
Q_3 ($^5T_{2g}$)	0.018	0.017	0.325

Table S4: Second-order diagonal coefficients $\gamma_i^{(\alpha)}$ for VCHAM-B3LYP*, given in eV. Both FC adiabatic and diabatic state labels, the latter in parantheses, are given. Note that the S_4–S_6 ($^1T_{2g}$) states were not used in the QD simulations but are reported here for completeness.

State	$\gamma_{13}^{(\alpha)}$	$\gamma_{14}^{(\alpha)}$	$\gamma_{15}^{(\alpha)}$
S_0 (^{1}GS)	0.007	0.005	−0.013
S_1 ($^1T_{1g}$)	0.010	0.008	−0.011
S_2 ($^1T_{1g}$)	0.011	0.018	−0.011
S_3 ($^1T_{1g}$)	0.021	0.011	−0.011
S_4 ($^1T_{2g}$)	0.013	0.013	N/A
S_5 ($^1T_{2g}$)	0.009	0.009	N/A
S_6 ($^1T_{2g}$)	0.002	0.000	N/A
T_1 ($^3T_{1g}$)	0.010	0.006	−0.011
T_2 ($^3T_{1g}$)	0.010	0.006	−0.011
T_3 ($^3T_{1g}$)	0.020	0.021	−0.011
T_4 ($^3T_{2g}$)	0.017	0.017	−0.011
T_5 ($^3T_{2g}$)	0.017	0.018	−0.011
T_6 ($^3T_{2g}$)	0.007	0.004	−0.011
Q_1 ($^5T_{2g}$)	0.019	0.018	−0.008
Q_2 ($^5T_{2g}$)	0.022	0.021	−0.006
Q_3 ($^5T_{2g}$)	0.020	0.019	−0.006

Table S5: First-order off-diagonal coefficients $\lambda_i^{(\alpha\beta)}$ for VCHAM-B3LYP*, given in eV. Both FC adiabatic and diabatic state labels, the latter in parantheses, are given. Note that the S_4–S_6 ($^1T_{2g}$) states were not used in the QD simulations but are reported here for completeness.

State	$\lambda_{13}^{(\alpha\beta)}$	$\lambda_{14}^{(\alpha\beta)}$	$\lambda_{15}^{(\alpha\beta)}$
S_1 ($^1T_{1g}$) - S_2 ($^1T_{1g}$)	−0.040	−0.032	0.000
S_1 ($^1T_{1g}$) - S_3 ($^1T_{1g}$)	0.064	0.007	0.000
S_1 ($^1T_{1g}$) - S_4 ($^1T_{2g}$)	0.082	0.080	0.000
S_1 ($^1T_{1g}$) - S_5 ($^1T_{2g}$)	0.000	0.000	0.000
S_1 ($^1T_{1g}$) - S_6 ($^1T_{2g}$)	0.000	0.000	0.000
S_2 ($^1T_{1g}$) - S_3 ($^1T_{1g}$)	0.050	0.079	0.000
S_2 ($^1T_{1g}$) - S_4 ($^1T_{2g}$)	0.000	0.000	0.000
S_2 ($^1T_{1g}$) - S_5 ($^1T_{2g}$)	0.073	0.078	0.000
S_2 ($^1T_{1g}$) - S_6 ($^1T_{2g}$)	0.000	0.000	0.000
S_3 ($^1T_{1g}$) - S_4 ($^1T_{2g}$)	0.000	0.000	0.000
S_3 ($^1T_{1g}$) - S_5 ($^1T_{2g}$)	0.073	0.091	0.000
S_3 ($^1T_{1g}$) - S_6 ($^1T_{2g}$)	−0.038	−0.011	0.000
S_4 ($^1T_{2g}$) - S_5 ($^1T_{2g}$)	0.025	0.020	0.000
S_4 ($^1T_{2g}$) - S_6 ($^1T_{2g}$)	0.074	0.044	0.000
S_5 ($^1T_{2g}$) - S_6 ($^1T_{2g}$)	0.045	0.050	0.000
T_1 ($^3T_{1g}$) - T_2 ($^3T_{1g}$)	0.008	−0.008	0.000
T_1 ($^3T_{1g}$) - T_3 ($^3T_{1g}$)	0.018	−0.029	0.000
T_1 ($^3T_{1g}$) - T_4 ($^3T_{2g}$)	0.121	0.115	0.000
T_1 ($^3T_{1g}$) - T_5 ($^3T_{2g}$)	0.000	0.000	0.000
T_1 ($^3T_{1g}$) - T_6 ($^3T_{2g}$)	0.000	0.000	0.000
T_2 ($^3T_{1g}$) - T_3 ($^3T_{1g}$)	−0.098	−0.079	0.000
T_2 ($^3T_{1g}$) - T_4 ($^3T_{2g}$)	0.000	0.000	0.000
T_2 ($^3T_{1g}$) - T_5 ($^3T_{2g}$)	0.074	0.085	0.000
T_2 ($^3T_{1g}$) - T_6 ($^3T_{2g}$)	0.000	0.000	0.000
T_3 ($^3T_{1g}$) - T_4 ($^3T_{2g}$)	0.000	0.000	0.000
T_3 ($^3T_{1g}$) - T_5 ($^3T_{2g}$)	−0.017	−0.017	0.000
T_3 ($^3T_{1g}$) - T_6 ($^3T_{2g}$)	0.054	0.054	0.000
T_4 ($^3T_{2g}$) - T_5 ($^3T_{2g}$)	−0.010	0.010	0.000
T_4 ($^3T_{2g}$) - T_6 ($^3T_{2g}$)	−0.016	0.022	0.000
T_5 ($^3T_{2g}$) - T_6 ($^3T_{2g}$)	0.087	0.072	0.000
Q_1 ($^5T_{2g}$) - Q_2 ($^5T_{2g}$)	−0.001	0.013	0.000
Q_1 ($^5T_{2g}$) - Q_3 ($^5T_{2g}$)	0.001	0.013	0.000
Q_2 ($^5T_{2g}$) - Q_3 ($^5T_{2g}$)	0.013	0.000	0.000

Table S6: First-order diagonal coefficients $\kappa_i^{(\alpha)}$ for LVC-B3LYP* ($\geq$ 0.001 eV) singlet states, given in eV.

Mode	$\kappa_i^{(S_0,GS)}$	$\kappa_i^{(S_1,{}^1T_{1g})}$	$\kappa_i^{(S_2,{}^1T_{1g})}$	$\kappa_i^{(S_3,{}^1T_{1g})}$
ν_4	0.000	0.000	0.000	0.001
ν_6	0.000	0.000	0.000	0.002
ν_{13}	0.000	0.000	0.000	0.064
ν_{14}	0.000	0.033	0.000	0.001
ν_{15}	0.030	0.201	0.201	0.201
ν_{16}	0.000	0.000	0.000	0.001
ν_{20}	0.000	0.000	0.001	0.000
ν_{23}	0.000	0.006	0.000	0.000
ν_{24}	0.000	0.002	0.000	0.000
ν_{40}	0.000	0.006	0.000	0.000
ν_{41}	0.000	0.000	0.000	0.013
ν_{46}	0.003	0.000	0.003	0.004

Table S7: First-order diagonal coefficients $\kappa_i^{(\alpha)}$ for LVC-B3LYP* (≥ 0.001 eV) triplet states, given in eV.

Mode	$\kappa_i^{(T_1,{}^3T_{1g})}$	$\kappa_i^{(T_2,{}^3T_{1g})}$	$\kappa_i^{(T_3,{}^3T_{1g})}$	$\kappa_i^{(T_4,{}^3T_{2g})}$	$\kappa_i^{(T_5,{}^3T_{2g})}$	$\kappa_i^{(T_6,{}^3T_{2g})}$
ν_4	0.000	0.000	0.000	0.000	0.000	0.001
ν_6	0.000	0.000	0.002	0.000	0.000	0.003
ν_{13}	0.000	0.000	0.069	0.000	0.000	0.072
ν_{14}	0.027	0.000	0.000	0.000	0.007	0.000
ν_{15}	0.214	0.214	0.214	0.209	0.209	0.209
ν_{16}	0.000	0.000	0.002	0.000	0.000	0.000
ν_{19}	0.000	0.001	0.000	0.001	0.000	0.000
ν_{20}	0.000	0.000	0.000	0.002	0.000	0.000
ν_{22}	0.000	0.000	0.000	0.001	0.000	0.000
ν_{23}	0.006	0.000	0.000	0.007	0.000	0.000
ν_{24}	0.003	0.000	0.000	0.003	0.000	0.000
ν_{37}	0.000	0.000	0.000	0.000	0.002	0.000
ν_{40}	0.006	0.000	0.000	0.000	0.002	0.000
ν_{41}	0.000	0.000	0.016	0.000	0.000	0.011
ν_{46}	0.000	0.004	0.006	0.007	0.003	0.000
ν_{50}	0.000	0.000	0.000	0.002	0.001	0.002

Table S8: First-order diagonal coefficients $\kappa_i^{(\alpha)}$ for LVC-B3LYP* ($\geq$ 0.001 eV) quintet states, given in eV.

Mode	$\kappa_i^{(Q_1,{}^5T_{2g})}$	$\kappa_i^{(Q_2,{}^5T_{2g})}$	$\kappa_i^{(Q_3,{}^5T_{2g})}$
ν_4	0.000	0.000	0.004
ν_{13}	0.006	0.000	0.008
ν_{14}	0.006	0.000	0.007
ν_{15}	0.389	0.389	0.391
ν_{16}	0.000	0.000	0.000
ν_{24}	0.000	0.010	0.002
ν_{39}	0.000	0.000	0.003
ν_{40}	0.004	0.000	0.005
ν_{41}	0.004	0.000	0.006
ν_{46}	0.004	0.000	0.004
ν_{47}	0.003	0.000	0.004
ν_{50}	0.000	0.002	0.000
ν_{51}	0.014	0.014	0.014

First-order off-diagonal coefficients $\lambda_i^{(\alpha\beta)}$ for LVC-B3LYP* ($\geq$ 0.001 eV) singlet states:

$i = \nu_{13}$, $\alpha = \mathrm{S}_2$ ($^1\mathrm{T}_{1\mathrm{g}}$), $\beta = \mathrm{S}_4$ ($^1\mathrm{T}_{1\mathrm{g}}$): 0.004 eV
$i = \nu_{14}$, $\alpha = \mathrm{S}_2$ ($^1\mathrm{T}_{1\mathrm{g}}$), $\beta = \mathrm{S}_3$ ($^1\mathrm{T}_{1\mathrm{g}}$): 0.017 eV
$i = \nu_{14}$, $\alpha = \mathrm{S}_3$ ($^1\mathrm{T}_{1\mathrm{g}}$), $\beta = \mathrm{S}_4$ ($^1\mathrm{T}_{1\mathrm{g}}$): 0.013 eV
$i = \nu_{16}$, $\alpha = \mathrm{S}_3$ ($^1\mathrm{T}_{1\mathrm{g}}$), $\beta = \mathrm{S}_4$ ($^1\mathrm{T}_{1\mathrm{g}}$): 0.001 eV
$i = \nu_{22}$, $\alpha = \mathrm{S}_2$ ($^1\mathrm{T}_{1\mathrm{g}}$), $\beta = \mathrm{S}_3$ ($^1\mathrm{T}_{1\mathrm{g}}$): 0.004 eV
$i = \nu_{22}$, $\alpha = \mathrm{S}_2$ ($^1\mathrm{T}_{1\mathrm{g}}$), $\beta = \mathrm{S}_4$ ($^1\mathrm{T}_{1\mathrm{g}}$): 0.005 eV
$i = \nu_{22}$, $\alpha = \mathrm{S}_3$ ($^1\mathrm{T}_{1\mathrm{g}}$), $\beta = \mathrm{S}_4$ ($^1\mathrm{T}_{1\mathrm{g}}$): 0.001 eV
$i = \nu_{23}$, $\alpha = \mathrm{S}_2$ ($^1\mathrm{T}_{1\mathrm{g}}$), $\beta = \mathrm{S}_4$ ($^1\mathrm{T}_{1\mathrm{g}}$): 0.003 eV
$i = \nu_{23}$, $\alpha = \mathrm{S}_3$ ($^1\mathrm{T}_{1\mathrm{g}}$), $\beta = \mathrm{S}_4$ ($^1\mathrm{T}_{1\mathrm{g}}$): 0.002 eV
$i = \nu_{39}$, $\alpha = \mathrm{S}_2$ ($^1\mathrm{T}_{1\mathrm{g}}$), $\beta = \mathrm{S}_3$ ($^1\mathrm{T}_{1\mathrm{g}}$): 0.001 eV
$i = \nu_{40}$, $\alpha = \mathrm{S}_2$ ($^1\mathrm{T}_{1\mathrm{g}}$), $\beta = \mathrm{S}_4$ ($^1\mathrm{T}_{1\mathrm{g}}$): 0.009 eV
$i = \nu_{47}$, $\alpha = \mathrm{S}_2$ ($^1\mathrm{T}_{1\mathrm{g}}$), $\beta = \mathrm{S}_4$ ($^1\mathrm{T}_{1\mathrm{g}}$): 0.001 eV

First-order off-diagonal coefficients $\lambda_i^{(\alpha\beta)}$ for LVC-B3LYP* ($\geq$ 0.001 eV) triplet states:

$i = \nu_1$, $\alpha = \mathrm{T}_1$ ($^3\mathrm{T}_{1\mathrm{g}}$, $\beta = \mathrm{T}_4$ ($^3\mathrm{T}_{2\mathrm{g}}$): 0.002 eV
$i = \nu_2$, $\alpha = \mathrm{T}_2$ ($^3\mathrm{T}_{1\mathrm{g}}$, $\beta = \mathrm{T}_4$ ($^3\mathrm{T}_{2\mathrm{g}}$): 0.002 eV
$i = \nu_2$, $\alpha = \mathrm{T}_3$ ($^3\mathrm{T}_{1\mathrm{g}}$, $\beta = \mathrm{T}_4$ ($^3\mathrm{T}_{2\mathrm{g}}$): 0.005 eV
$i = \nu_3$, $\alpha = \mathrm{T}_1$ ($^3\mathrm{T}_{1\mathrm{g}}$, $\beta = \mathrm{T}_6$ ($^3\mathrm{T}_{2\mathrm{g}}$): 0.001 eV
$i = \nu_4$, $\alpha = \mathrm{T}_1$ ($^3\mathrm{T}_{1\mathrm{g}}$, $\beta = \mathrm{T}_4$ ($^3\mathrm{T}_{2\mathrm{g}}$): 0.001 eV
$i = \nu_4$, $\alpha = \mathrm{T}_1$ ($^3\mathrm{T}_{1\mathrm{g}}$, $\beta = \mathrm{T}_5$ ($^3\mathrm{T}_{2\mathrm{g}}$): 0.002 eV
$i = \nu_4$, $\alpha = \mathrm{T}_2$ ($^3\mathrm{T}_{1\mathrm{g}}$, $\beta = \mathrm{T}_6$ ($^3\mathrm{T}_{2\mathrm{g}}$): 0.006 eV
$i = \nu_4$, $\alpha = \mathrm{T}_3$ ($^3\mathrm{T}_{1\mathrm{g}}$, $\beta = \mathrm{T}_4$ ($^3\mathrm{T}_{2\mathrm{g}}$): 0.011 eV
$i = \nu_4$, $\alpha = \mathrm{T}_4$ ($^3\mathrm{T}_{2\mathrm{g}}$, $\beta = \mathrm{T}_6$ ($^3\mathrm{T}_{2\mathrm{g}}$): 0.003 eV
$i = \nu_4$, $\alpha = \mathrm{T}_5$ ($^3\mathrm{T}_{2\mathrm{g}}$, $\beta = \mathrm{T}_6$ ($^3\mathrm{T}_{2\mathrm{g}}$): 0.002 eV
$i = \nu_5$, $\alpha = \mathrm{T}_1$ ($^3\mathrm{T}_{1\mathrm{g}}$, $\beta = \mathrm{T}_4$ ($^3\mathrm{T}_{2\mathrm{g}}$): 0.005 eV
$i = \nu_5$, $\alpha = \mathrm{T}_1$ ($^3\mathrm{T}_{1\mathrm{g}}$, $\beta = \mathrm{T}_6$ ($^3\mathrm{T}_{2\mathrm{g}}$): 0.002 eV
$i = \nu_5$, $\alpha = \mathrm{T}_2$ ($^3\mathrm{T}_{1\mathrm{g}}$, $\beta = \mathrm{T}_6$ ($^3\mathrm{T}_{2\mathrm{g}}$): 0.002 eV
$i = \nu_5$, $\alpha = \mathrm{T}_3$ ($^3\mathrm{T}_{1\mathrm{g}}$, $\beta = \mathrm{T}_4$ ($^3\mathrm{T}_{2\mathrm{g}}$): 0.002 eV
$i = \nu_5$, $\alpha = \mathrm{T}_3$ ($^3\mathrm{T}_{1\mathrm{g}}$, $\beta = \mathrm{T}_6$ ($^3\mathrm{T}_{2\mathrm{g}}$): 0.006 eV
$i = \nu_5$, $\alpha = \mathrm{T}_5$ ($^3\mathrm{T}_{2\mathrm{g}}$, $\beta = \mathrm{T}_6$ ($^3\mathrm{T}_{2\mathrm{g}}$): 0.002 eV
$i = \nu_6$, $\alpha = \mathrm{T}_1$ ($^3\mathrm{T}_{1\mathrm{g}}$, $\beta = \mathrm{T}_3$ ($^3\mathrm{T}_{1\mathrm{g}}$): 0.001 eV
$i = \nu_6$, $\alpha = \mathrm{T}_1$ ($^3\mathrm{T}_{1\mathrm{g}}$, $\beta = \mathrm{T}_5$ ($^3\mathrm{T}_{2\mathrm{g}}$): 0.003 eV
$i = \nu_6$, $\alpha = \mathrm{T}_1$ ($^3\mathrm{T}_{1\mathrm{g}}$, $\beta = \mathrm{T}_6$ ($^3\mathrm{T}_{2\mathrm{g}}$): 0.002 eV
$i = \nu_6$, $\alpha = \mathrm{T}_3$ ($^3\mathrm{T}_{1\mathrm{g}}$, $\beta = \mathrm{T}_4$ ($^3\mathrm{T}_{2\mathrm{g}}$): 0.002 eV

$i = \nu_6$, $\alpha = \mathrm{T}_3$ ($^3\mathrm{T}_{1g}$, $\beta = \mathrm{T}_5$ ($^3\mathrm{T}_{2g}$): 0.007 eV
$i = \nu_6$, $\alpha = \mathrm{T}_3$ ($^3\mathrm{T}_{1g}$, $\beta = \mathrm{T}_6$ ($^3\mathrm{T}_{2g}$): 0.004 eV
$i = \nu_7$, $\alpha = \mathrm{T}_1$ ($^3\mathrm{T}_{1g}$, $\beta = \mathrm{T}_5$ ($^3\mathrm{T}_{2g}$): 0.001 eV
$i = \nu_8$, $\alpha = \mathrm{T}_1$ ($^3\mathrm{T}_{1g}$, $\beta = \mathrm{T}_4$ ($^3\mathrm{T}_{2g}$): 0.002 eV
$i = \nu_9$, $\alpha = \mathrm{T}_2$ ($^3\mathrm{T}_{1g}$, $\beta = \mathrm{T}_6$ ($^3\mathrm{T}_{2g}$): 0.003 eV
$i = \nu_{10}$, $\alpha = \mathrm{T}_1$ ($^3\mathrm{T}_{1g}$, $\beta = \mathrm{T}_5$ ($^3\mathrm{T}_{2g}$): 0.006 eV
$i = \nu_{10}$, $\alpha = \mathrm{T}_2$ ($^3\mathrm{T}_{1g}$, $\beta = \mathrm{T}_4$ ($^3\mathrm{T}_{2g}$): 0.005 eV
$i = \nu_{10}$, $\alpha = \mathrm{T}_2$ ($^3\mathrm{T}_{1g}$, $\beta = \mathrm{T}_5$ ($^3\mathrm{T}_{2g}$): 0.002 eV
$i = \nu_{10}$, $\alpha = \mathrm{T}_3$ ($^3\mathrm{T}_{1g}$, $\beta = \mathrm{T}_5$ ($^3\mathrm{T}_{2g}$): 0.005 eV
$i = \nu_{11}$, $\alpha = \mathrm{T}_2$ ($^3\mathrm{T}_{1g}$, $\beta = \mathrm{T}_4$ ($^3\mathrm{T}_{2g}$): 0.008 eV
$i = \nu_{11}$, $\alpha = \mathrm{T}_3$ ($^3\mathrm{T}_{1g}$, $\beta = \mathrm{T}_5$ ($^3\mathrm{T}_{2g}$): 0.008 eV
$i = \nu_{11}$, $\alpha = \mathrm{T}_3$ ($^3\mathrm{T}_{1g}$, $\beta = \mathrm{T}_6$ ($^3\mathrm{T}_{2g}$): 0.001 eV
$i = \nu_{12}$, $\alpha = \mathrm{T}_1$ ($^3\mathrm{T}_{1g}$, $\beta = \mathrm{T}_4$ ($^3\mathrm{T}_{2g}$): 0.002 eV
$i = \nu_{12}$, $\alpha = \mathrm{T}_3$ ($^3\mathrm{T}_{1g}$, $\beta = \mathrm{T}_6$ ($^3\mathrm{T}_{2g}$): 0.003 eV
$i = \nu_{13}$, $\alpha = \mathrm{T}_1$ ($^3\mathrm{T}_{1g}$, $\beta = \mathrm{T}_2$ ($^3\mathrm{T}_{1g}$): 0.051 eV
$i = \nu_{13}$, $\alpha = \mathrm{T}_1$ ($^3\mathrm{T}_{1g}$, $\beta = \mathrm{T}_3$ ($^3\mathrm{T}_{1g}$): 0.007 eV
$i = \nu_{13}$, $\alpha = \mathrm{T}_1$ ($^3\mathrm{T}_{1g}$, $\beta = \mathrm{T}_5$ ($^3\mathrm{T}_{2g}$): 0.009 eV
$i = \nu_{13}$, $\alpha = \mathrm{T}_1$ ($^3\mathrm{T}_{1g}$, $\beta = \mathrm{T}_6$ ($^3\mathrm{T}_{2g}$): 0.013 eV
$i = \nu_{13}$, $\alpha = \mathrm{T}_2$ ($^3\mathrm{T}_{1g}$, $\beta = \mathrm{T}_3$ ($^3\mathrm{T}_{1g}$): 0.003 eV
$i = \nu_{13}$, $\alpha = \mathrm{T}_2$ ($^3\mathrm{T}_{1g}$, $\beta = \mathrm{T}_4$ ($^3\mathrm{T}_{2g}$): 0.001 eV
$i = \nu_{13}$, $\alpha = \mathrm{T}_2$ ($^3\mathrm{T}_{1g}$, $\beta = \mathrm{T}_5$ ($^3\mathrm{T}_{2g}$): 0.095 eV
$i = \nu_{13}$, $\alpha = \mathrm{T}_2$ ($^3\mathrm{T}_{1g}$, $\beta = \mathrm{T}_6$ ($^3\mathrm{T}_{2g}$): 0.017 eV
$i = \nu_{13}$, $\alpha = \mathrm{T}_3$ ($^3\mathrm{T}_{1g}$, $\beta = \mathrm{T}_6$ ($^3\mathrm{T}_{2g}$): 0.004 eV
$i = \nu_{13}$, $\alpha = \mathrm{T}_4$ ($^3\mathrm{T}_{2g}$, $\beta = \mathrm{T}_5$ ($^3\mathrm{T}_{2g}$): 0.026 eV
$i = \nu_{14}$, $\alpha = \mathrm{T}_1$ ($^3\mathrm{T}_{1g}$, $\beta = \mathrm{T}_3$ ($^3\mathrm{T}_{1g}$): 0.055 eV
$i = \nu_{14}$, $\alpha = \mathrm{T}_1$ ($^3\mathrm{T}_{1g}$, $\beta = \mathrm{T}_6$ ($^3\mathrm{T}_{2g}$): 0.063 eV
$i = \nu_{14}$, $\alpha = \mathrm{T}_2$ ($^3\mathrm{T}_{1g}$, $\beta = \mathrm{T}_3$ ($^3\mathrm{T}_{1g}$): 0.012 eV
$i = \nu_{14}$, $\alpha = \mathrm{T}_2$ ($^3\mathrm{T}_{1g}$, $\beta = \mathrm{T}_4$ ($^3\mathrm{T}_{2g}$): 0.002 eV
$i = \nu_{14}$, $\alpha = \mathrm{T}_2$ ($^3\mathrm{T}_{1g}$, $\beta = \mathrm{T}_6$ ($^3\mathrm{T}_{2g}$): 0.003 eV
$i = \nu_{14}$, $\alpha = \mathrm{T}_3$ ($^3\mathrm{T}_{1g}$, $\beta = \mathrm{T}_4$ ($^3\mathrm{T}_{2g}$): 0.072 eV
$i = \nu_{14}$, $\alpha = \mathrm{T}_3$ ($^3\mathrm{T}_{1g}$, $\beta = \mathrm{T}_5$ ($^3\mathrm{T}_{2g}$): 0.002 eV
$i = \nu_{14}$, $\alpha = \mathrm{T}_3$ ($^3\mathrm{T}_{1g}$, $\beta = \mathrm{T}_6$ ($^3\mathrm{T}_{2g}$): 0.001 eV
$i = \nu_{14}$, $\alpha = \mathrm{T}_4$ ($^3\mathrm{T}_{2g}$, $\beta = \mathrm{T}_6$ ($^3\mathrm{T}_{2g}$): 0.057 eV
$i = \nu_{15}$, $\alpha = \mathrm{T}_2$ ($^3\mathrm{T}_{1g}$, $\beta = \mathrm{T}_4$ ($^3\mathrm{T}_{2g}$): 0.005 eV
$i = \nu_{15}$, $\alpha = \mathrm{T}_2$ ($^3\mathrm{T}_{1g}$, $\beta = \mathrm{T}_5$ ($^3\mathrm{T}_{2g}$): 0.006 eV
$i = \nu_{15}$, $\alpha = \mathrm{T}_2$ ($^3\mathrm{T}_{1g}$, $\beta = \mathrm{T}_6$ ($^3\mathrm{T}_{2g}$): 0.005 eV

$i = \nu_{15}$, $\alpha =$ T$_3$ (^{3}T$_{1g}$, $\beta =$ T$_4$ (^{3}T$_{2g}$): 0.002 eV
$i = \nu_{15}$, $\alpha =$ T$_3$ (^{3}T$_{1g}$, $\beta =$ T$_5$ (^{3}T$_{2g}$): 0.003 eV
$i = \nu_{15}$, $\alpha =$ T$_3$ (^{3}T$_{1g}$, $\beta =$ T$_6$ (^{3}T$_{2g}$): 0.003 eV
$i = \nu_{16}$, $\alpha =$ T$_2$ (^{3}T$_{1g}$, $\beta =$ T$_5$ (^{3}T$_{2g}$): 0.002 eV
$i = \nu_{16}$, $\alpha =$ T$_2$ (^{3}T$_{1g}$, $\beta =$ T$_6$ (^{3}T$_{2g}$): 0.005 eV
$i = \nu_{17}$, $\alpha =$ T$_2$ (^{3}T$_{1g}$, $\beta =$ T$_6$ (^{3}T$_{2g}$): 0.007 eV
$i = \nu_{18}$, $\alpha =$ T$_1$ (^{3}T$_{1g}$, $\beta =$ T$_4$ (^{3}T$_{2g}$): 0.005 eV
$i = \nu_{18}$, $\alpha =$ T$_3$ (^{3}T$_{1g}$, $\beta =$ T$_5$ (^{3}T$_{2g}$): 0.005 eV
$i = \nu_{19}$, $\alpha =$ T$_1$ (^{3}T$_{1g}$, $\beta =$ T$_4$ (^{3}T$_{2g}$): 0.003 eV
$i = \nu_{19}$, $\alpha =$ T$_3$ (^{3}T$_{1g}$, $\beta =$ T$_4$ (^{3}T$_{2g}$): 0.002 eV
$i = \nu_{19}$, $\alpha =$ T$_3$ (^{3}T$_{1g}$, $\beta =$ T$_5$ (^{3}T$_{2g}$): 0.001 eV
$i = \nu_{19}$, $\alpha =$ T$_4$ (^{3}T$_{2g}$, $\beta =$ T$_5$ (^{3}T$_{2g}$): 0.001 eV
$i = \nu_{20}$, $\alpha =$ T$_1$ (^{3}T$_{1g}$, $\beta =$ T$_5$ (^{3}T$_{2g}$): 0.006 eV
$i = \nu_{20}$, $\alpha =$ T$_2$ (^{3}T$_{1g}$, $\beta =$ T$_4$ (^{3}T$_{2g}$): 0.003 eV
$i = \nu_{20}$, $\alpha =$ T$_3$ (^{3}T$_{1g}$, $\beta =$ T$_4$ (^{3}T$_{2g}$): 0.005 eV
$i = \nu_{20}$, $\alpha =$ T$_3$ (^{3}T$_{1g}$, $\beta =$ T$_5$ (^{3}T$_{2g}$): 0.004 eV
$i = \nu_{21}$, $\alpha =$ T$_1$ (^{3}T$_{1g}$, $\beta =$ T$_4$ (^{3}T$_{2g}$): 0.006 eV
$i = \nu_{21}$, $\alpha =$ T$_2$ (^{3}T$_{1g}$, $\beta =$ T$_5$ (^{3}T$_{2g}$): 0.004 eV
$i = \nu_{21}$, $\alpha =$ T$_3$ (^{3}T$_{1g}$, $\beta =$ T$_4$ (^{3}T$_{2g}$): 0.005 eV
$i = \nu_{22}$, $\alpha =$ T$_1$ (^{3}T$_{1g}$, $\beta =$ T$_4$ (^{3}T$_{2g}$): 0.006 eV
$i = \nu_{22}$, $\alpha =$ T$_3$ (^{3}T$_{1g}$, $\beta =$ T$_4$ (^{3}T$_{2g}$): 0.002 eV
$i = \nu_{23}$, $\alpha =$ T$_1$ (^{3}T$_{1g}$, $\beta =$ T$_2$ (^{3}T$_{1g}$): 0.002 eV
$i = \nu_{23}$, $\alpha =$ T$_2$ (^{3}T$_{1g}$, $\beta =$ T$_5$ (^{3}T$_{2g}$): 0.006 eV
$i = \nu_{23}$, $\alpha =$ T$_2$ (^{3}T$_{1g}$, $\beta =$ T$_6$ (^{3}T$_{2g}$): 0.003 eV
$i = \nu_{23}$, $\alpha =$ T$_3$ (^{3}T$_{1g}$, $\beta =$ T$_5$ (^{3}T$_{2g}$): 0.002 eV
$i = \nu_{23}$, $\alpha =$ T$_3$ (^{3}T$_{1g}$, $\beta =$ T$_6$ (^{3}T$_{2g}$): 0.004 eV
$i = \nu_{23}$, $\alpha =$ T$_4$ (^{3}T$_{2g}$, $\beta =$ T$_5$ (^{3}T$_{2g}$): 0.002 eV
$i = \nu_{23}$, $\alpha =$ T$_5$ (^{3}T$_{2g}$, $\beta =$ T$_6$ (^{3}T$_{2g}$): 0.002 eV
$i = \nu_{24}$, $\alpha =$ T$_1$ (^{3}T$_{1g}$, $\beta =$ T$_6$ (^{3}T$_{2g}$): 0.014 eV
$i = \nu_{24}$, $\alpha =$ T$_3$ (^{3}T$_{1g}$, $\beta =$ T$_5$ (^{3}T$_{2g}$): 0.007 eV
$i = \nu_{24}$, $\alpha =$ T$_5$ (^{3}T$_{2g}$, $\beta =$ T$_6$ (^{3}T$_{2g}$): 0.005 eV
$i = \nu_{25}$, $\alpha =$ T$_1$ (^{3}T$_{1g}$, $\beta =$ T$_5$ (^{3}T$_{2g}$): 0.005 eV
$i = \nu_{25}$, $\alpha =$ T$_2$ (^{3}T$_{1g}$, $\beta =$ T$_5$ (^{3}T$_{2g}$): 0.003 eV
$i = \nu_{26}$, $\alpha =$ T$_2$ (^{3}T$_{1g}$, $\beta =$ T$_6$ (^{3}T$_{2g}$): 0.001 eV
$i = \nu_{28}$, $\alpha =$ T$_2$ (^{3}T$_{1g}$, $\beta =$ T$_4$ (^{3}T$_{2g}$): 0.005 eV
$i = \nu_{29}$, $\alpha =$ T$_1$ (^{3}T$_{1g}$, $\beta =$ T$_5$ (^{3}T$_{2g}$): 0.019 eV

$i = \nu_{29}$, $\alpha = \mathrm{T}_2$ ($^3\mathrm{T}_{1g}$, $\beta = \mathrm{T}_4$ ($^3\mathrm{T}_{2g}$): 0.015 eV
$i = \nu_{29}$, $\alpha = \mathrm{T}_2$ ($^3\mathrm{T}_{1g}$, $\beta = \mathrm{T}_5$ ($^3\mathrm{T}_{2g}$): 0.001 eV
$i = \nu_{29}$, $\alpha = \mathrm{T}_2$ ($^3\mathrm{T}_{1g}$, $\beta = \mathrm{T}_6$ ($^3\mathrm{T}_{2g}$): 0.008 eV
$i = \nu_{30}$, $\alpha = \mathrm{T}_1$ ($^3\mathrm{T}_{1g}$, $\beta = \mathrm{T}_4$ ($^3\mathrm{T}_{2g}$): 0.005 eV
$i = \nu_{30}$, $\alpha = \mathrm{T}_1$ ($^3\mathrm{T}_{1g}$, $\beta = \mathrm{T}_5$ ($^3\mathrm{T}_{2g}$): 0.012 eV
$i = \nu_{30}$, $\alpha = \mathrm{T}_1$ ($^3\mathrm{T}_{1g}$, $\beta = \mathrm{T}_6$ ($^3\mathrm{T}_{2g}$): 0.001 eV
$i = \nu_{30}$, $\alpha = \mathrm{T}_2$ ($^3\mathrm{T}_{1g}$, $\beta = \mathrm{T}_5$ ($^3\mathrm{T}_{2g}$): 0.001 eV
$i = \nu_{30}$, $\alpha = \mathrm{T}_3$ ($^3\mathrm{T}_{1g}$, $\beta = \mathrm{T}_4$ ($^3\mathrm{T}_{2g}$): 0.004 eV
$i = \nu_{31}$, $\alpha = \mathrm{T}_2$ ($^3\mathrm{T}_{1g}$, $\beta = \mathrm{T}_4$ ($^3\mathrm{T}_{2g}$): 0.005 eV
$i = \nu_{31}$, $\alpha = \mathrm{T}_2$ ($^3\mathrm{T}_{1g}$, $\beta = \mathrm{T}_6$ ($^3\mathrm{T}_{2g}$): 0.002 eV
$i = \nu_{32}$, $\alpha = \mathrm{T}_1$ ($^3\mathrm{T}_{1g}$, $\beta = \mathrm{T}_4$ ($^3\mathrm{T}_{2g}$): 0.006 eV
$i = \nu_{32}$, $\alpha = \mathrm{T}_1$ ($^3\mathrm{T}_{1g}$, $\beta = \mathrm{T}_6$ ($^3\mathrm{T}_{2g}$): 0.004 eV
$i = \nu_{32}$, $\alpha = \mathrm{T}_2$ ($^3\mathrm{T}_{1g}$, $\beta = \mathrm{T}_6$ ($^3\mathrm{T}_{2g}$): 0.002 eV
$i = \nu_{32}$, $\alpha = \mathrm{T}_3$ ($^3\mathrm{T}_{1g}$, $\beta = \mathrm{T}_4$ ($^3\mathrm{T}_{2g}$): 0.002 eV
$i = \nu_{33}$, $\alpha = \mathrm{T}_1$ ($^3\mathrm{T}_{1g}$, $\beta = \mathrm{T}_5$ ($^3\mathrm{T}_{2g}$): 0.002 eV
$i = \nu_{33}$, $\alpha = \mathrm{T}_1$ ($^3\mathrm{T}_{1g}$, $\beta = \mathrm{T}_6$ ($^3\mathrm{T}_{2g}$): 0.006 eV
$i = \nu_{33}$, $\alpha = \mathrm{T}_2$ ($^3\mathrm{T}_{1g}$, $\beta = \mathrm{T}_4$ ($^3\mathrm{T}_{2g}$): 0.002 eV
$i = \nu_{33}$, $\alpha = \mathrm{T}_2$ ($^3\mathrm{T}_{1g}$, $\beta = \mathrm{T}_6$ ($^3\mathrm{T}_{2g}$): 0.002 eV
$i = \nu_{33}$, $\alpha = \mathrm{T}_3$ ($^3\mathrm{T}_{1g}$, $\beta = \mathrm{T}_6$ ($^3\mathrm{T}_{2g}$): 0.002 eV
$i = \nu_{34}$, $\alpha = \mathrm{T}_2$ ($^3\mathrm{T}_{1g}$, $\beta = \mathrm{T}_5$ ($^3\mathrm{T}_{2g}$): 0.004 eV
$i = \nu_{34}$, $\alpha = \mathrm{T}_3$ ($^3\mathrm{T}_{1g}$, $\beta = \mathrm{T}_4$ ($^3\mathrm{T}_{2g}$): 0.002 eV
$i = \nu_{35}$, $\alpha = \mathrm{T}_3$ ($^3\mathrm{T}_{1g}$, $\beta = \mathrm{T}_6$ ($^3\mathrm{T}_{2g}$): 0.004 eV
$i = \nu_{36}$, $\alpha = \mathrm{T}_2$ ($^3\mathrm{T}_{1g}$, $\beta = \mathrm{T}_4$ ($^3\mathrm{T}_{2g}$): 0.001 eV
$i = \nu_{36}$, $\alpha = \mathrm{T}_3$ ($^3\mathrm{T}_{1g}$, $\beta = \mathrm{T}_4$ ($^3\mathrm{T}_{2g}$): 0.005 eV
$i = \nu_{37}$, $\alpha = \mathrm{T}_1$ ($^3\mathrm{T}_{1g}$, $\beta = \mathrm{T}_5$ ($^3\mathrm{T}_{2g}$): 0.003 eV
$i = \nu_{37}$, $\alpha = \mathrm{T}_2$ ($^3\mathrm{T}_{1g}$, $\beta = \mathrm{T}_4$ ($^3\mathrm{T}_{2g}$): 0.002 eV
$i = \nu_{37}$, $\alpha = \mathrm{T}_2$ ($^3\mathrm{T}_{1g}$, $\beta = \mathrm{T}_5$ ($^3\mathrm{T}_{2g}$): 0.002 eV
$i = \nu_{37}$, $\alpha = \mathrm{T}_2$ ($^3\mathrm{T}_{1g}$, $\beta = \mathrm{T}_6$ ($^3\mathrm{T}_{2g}$): 0.035 eV
$i = \nu_{37}$, $\alpha = \mathrm{T}_3$ ($^3\mathrm{T}_{1g}$, $\beta = \mathrm{T}_4$ ($^3\mathrm{T}_{2g}$): 0.007 eV
$i = \nu_{37}$, $\alpha = \mathrm{T}_3$ ($^3\mathrm{T}_{1g}$, $\beta = \mathrm{T}_5$ ($^3\mathrm{T}_{2g}$): 0.008 eV
$i = \nu_{38}$, $\alpha = \mathrm{T}_1$ ($^3\mathrm{T}_{1g}$, $\beta = \mathrm{T}_5$ ($^3\mathrm{T}_{2g}$): 0.002 eV
$i = \nu_{38}$, $\alpha = \mathrm{T}_2$ ($^3\mathrm{T}_{1g}$, $\beta = \mathrm{T}_4$ ($^3\mathrm{T}_{2g}$): 0.002 eV
$i = \nu_{38}$, $\alpha = \mathrm{T}_3$ ($^3\mathrm{T}_{1g}$, $\beta = \mathrm{T}_4$ ($^3\mathrm{T}_{2g}$): 0.001 eV
$i = \nu_{39}$, $\alpha = \mathrm{T}_2$ ($^3\mathrm{T}_{1g}$, $\beta = \mathrm{T}_6$ ($^3\mathrm{T}_{2g}$): 0.013 eV
$i = \nu_{39}$, $\alpha = \mathrm{T}_3$ ($^3\mathrm{T}_{1g}$, $\beta = \mathrm{T}_4$ ($^3\mathrm{T}_{2g}$): 0.002 eV
$i = \nu_{39}$, $\alpha = \mathrm{T}_3$ ($^3\mathrm{T}_{1g}$, $\beta = \mathrm{T}_5$ ($^3\mathrm{T}_{2g}$): 0.011 eV

$i = \nu_{39}$, $\alpha = \mathrm{T}_3$ ($^3\mathrm{T}_{1g}$, $\beta = \mathrm{T}_6$ ($^3\mathrm{T}_{2g}$): 0.002 eV
$i = \nu_{40}$, $\alpha = \mathrm{T}_1$ ($^3\mathrm{T}_{1g}$, $\beta = \mathrm{T}_3$ ($^3\mathrm{T}_{1g}$): 0.012 eV
$i = \nu_{40}$, $\alpha = \mathrm{T}_1$ ($^3\mathrm{T}_{1g}$, $\beta = \mathrm{T}_4$ ($^3\mathrm{T}_{2g}$): 0.001 eV
$i = \nu_{40}$, $\alpha = \mathrm{T}_2$ ($^3\mathrm{T}_{1g}$, $\beta = \mathrm{T}_5$ ($^3\mathrm{T}_{2g}$): 0.025 eV
$i = \nu_{40}$, $\alpha = \mathrm{T}_3$ ($^3\mathrm{T}_{1g}$, $\beta = \mathrm{T}_5$ ($^3\mathrm{T}_{2g}$): 0.002 eV
$i = \nu_{40}$, $\alpha = \mathrm{T}_3$ ($^3\mathrm{T}_{1g}$, $\beta = \mathrm{T}_6$ ($^3\mathrm{T}_{2g}$): 0.001 eV
$i = \nu_{40}$, $\alpha = \mathrm{T}_4$ ($^3\mathrm{T}_{2g}$, $\beta = \mathrm{T}_5$ ($^3\mathrm{T}_{2g}$): 0.002 eV
$i = \nu_{40}$, $\alpha = \mathrm{T}_5$ ($^3\mathrm{T}_{2g}$, $\beta = \mathrm{T}_6$ ($^3\mathrm{T}_{2g}$): 0.001 eV
$i = \nu_{41}$, $\alpha = \mathrm{T}_1$ ($^3\mathrm{T}_{1g}$, $\beta = \mathrm{T}_2$ ($^3\mathrm{T}_{1g}$): 0.012 eV
$i = \nu_{41}$, $\alpha = \mathrm{T}_1$ ($^3\mathrm{T}_{1g}$, $\beta = \mathrm{T}_4$ ($^3\mathrm{T}_{2g}$): 0.002 eV
$i = \nu_{41}$, $\alpha = \mathrm{T}_2$ ($^3\mathrm{T}_{1g}$, $\beta = \mathrm{T}_4$ ($^3\mathrm{T}_{2g}$): 0.003 eV
$i = \nu_{41}$, $\alpha = \mathrm{T}_2$ ($^3\mathrm{T}_{1g}$, $\beta = \mathrm{T}_5$ ($^3\mathrm{T}_{2g}$): 0.023 eV
$i = \nu_{41}$, $\alpha = \mathrm{T}_5$ ($^3\mathrm{T}_{2g}$, $\beta = \mathrm{T}_6$ ($^3\mathrm{T}_{2g}$): 0.008 eV
$i = \nu_{42}$, $\alpha = \mathrm{T}_1$ ($^3\mathrm{T}_{1g}$, $\beta = \mathrm{T}_5$ ($^3\mathrm{T}_{2g}$): 0.001 eV
$i = \nu_{42}$, $\alpha = \mathrm{T}_2$ ($^3\mathrm{T}_{1g}$, $\beta = \mathrm{T}_6$ ($^3\mathrm{T}_{2g}$): 0.001 eV
$i = \nu_{43}$, $\alpha = \mathrm{T}_1$ ($^3\mathrm{T}_{1g}$, $\beta = \mathrm{T}_4$ ($^3\mathrm{T}_{2g}$): 0.002 eV
$i = \nu_{43}$, $\alpha = \mathrm{T}_2$ ($^3\mathrm{T}_{1g}$, $\beta = \mathrm{T}_4$ ($^3\mathrm{T}_{2g}$): 0.002 eV
$i = \nu_{43}$, $\alpha = \mathrm{T}_2$ ($^3\mathrm{T}_{1g}$, $\beta = \mathrm{T}_5$ ($^3\mathrm{T}_{2g}$): 0.003 eV
$i = \nu_{43}$, $\alpha = \mathrm{T}_2$ ($^3\mathrm{T}_{1g}$, $\beta = \mathrm{T}_6$ ($^3\mathrm{T}_{2g}$): 0.003 eV
$i = \nu_{43}$, $\alpha = \mathrm{T}_3$ ($^3\mathrm{T}_{1g}$, $\beta = \mathrm{T}_4$ ($^3\mathrm{T}_{2g}$): 0.005 eV
$i = \nu_{43}$, $\alpha = \mathrm{T}_3$ ($^3\mathrm{T}_{1g}$, $\beta = \mathrm{T}_5$ ($^3\mathrm{T}_{2g}$): 0.002 eV
$i = \nu_{44}$, $\alpha = \mathrm{T}_1$ ($^3\mathrm{T}_{1g}$, $\beta = \mathrm{T}_6$ ($^3\mathrm{T}_{2g}$): 0.004 eV
$i = \nu_{44}$, $\alpha = \mathrm{T}_2$ ($^3\mathrm{T}_{1g}$, $\beta = \mathrm{T}_4$ ($^3\mathrm{T}_{2g}$): 0.004 eV
$i = \nu_{44}$, $\alpha = \mathrm{T}_2$ ($^3\mathrm{T}_{1g}$, $\beta = \mathrm{T}_5$ ($^3\mathrm{T}_{2g}$): 0.005 eV
$i = \nu_{44}$, $\alpha = \mathrm{T}_3$ ($^3\mathrm{T}_{1g}$, $\beta = \mathrm{T}_5$ ($^3\mathrm{T}_{2g}$): 0.003 eV
$i = \nu_{45}$, $\alpha = \mathrm{T}_2$ ($^3\mathrm{T}_{1g}$, $\beta = \mathrm{T}_6$ ($^3\mathrm{T}_{2g}$): 0.005 eV
$i = \nu_{45}$, $\alpha = \mathrm{T}_3$ ($^3\mathrm{T}_{1g}$, $\beta = \mathrm{T}_4$ ($^3\mathrm{T}_{2g}$): 0.002 eV
$i = \nu_{45}$, $\alpha = \mathrm{T}_3$ ($^3\mathrm{T}_{1g}$, $\beta = \mathrm{T}_5$ ($^3\mathrm{T}_{2g}$): 0.004 eV
$i = \nu_{45}$, $\alpha = \mathrm{T}_3$ ($^3\mathrm{T}_{1g}$, $\beta = \mathrm{T}_6$ ($^3\mathrm{T}_{2g}$): 0.001 eV
$i = \nu_{46}$, $\alpha = \mathrm{T}_1$ ($^3\mathrm{T}_{1g}$, $\beta = \mathrm{T}_2$ ($^3\mathrm{T}_{1g}$): 0.001 eV
$i = \nu_{46}$, $\alpha = \mathrm{T}_1$ ($^3\mathrm{T}_{1g}$, $\beta = \mathrm{T}_4$ ($^3\mathrm{T}_{2g}$): 0.004 eV
$i = \nu_{46}$, $\alpha = \mathrm{T}_2$ ($^3\mathrm{T}_{1g}$, $\beta = \mathrm{T}_4$ ($^3\mathrm{T}_{2g}$): 0.005 eV
$i = \nu_{46}$, $\alpha = \mathrm{T}_2$ ($^3\mathrm{T}_{1g}$, $\beta = \mathrm{T}_5$ ($^3\mathrm{T}_{2g}$): 0.003 eV
$i = \nu_{46}$, $\alpha = \mathrm{T}_2$ ($^3\mathrm{T}_{1g}$, $\beta = \mathrm{T}_6$ ($^3\mathrm{T}_{2g}$): 0.018 eV
$i = \nu_{46}$, $\alpha = \mathrm{T}_3$ ($^3\mathrm{T}_{1g}$, $\beta = \mathrm{T}_4$ ($^3\mathrm{T}_{2g}$): 0.003 eV
$i = \nu_{46}$, $\alpha = \mathrm{T}_3$ ($^3\mathrm{T}_{1g}$, $\beta = \mathrm{T}_5$ ($^3\mathrm{T}_{2g}$): 0.018 eV

$i = \nu_{47}$, $\alpha = \mathrm{T}_1$ ($^3\mathrm{T}_{1g}$, $\beta = \mathrm{T}_3$ ($^3\mathrm{T}_{1g}$): 0.002 eV
$i = \nu_{47}$, $\alpha = \mathrm{T}_1$ ($^3\mathrm{T}_{1g}$, $\beta = \mathrm{T}_4$ ($^3\mathrm{T}_{2g}$): 0.005 eV
$i = \nu_{47}$, $\alpha = \mathrm{T}_1$ ($^3\mathrm{T}_{1g}$, $\beta = \mathrm{T}_5$ ($^3\mathrm{T}_{2g}$): 0.009 eV
$i = \nu_{47}$, $\alpha = \mathrm{T}_2$ ($^3\mathrm{T}_{1g}$, $\beta = \mathrm{T}_5$ ($^3\mathrm{T}_{2g}$): 0.004 eV
$i = \nu_{47}$, $\alpha = \mathrm{T}_2$ ($^3\mathrm{T}_{1g}$, $\beta = \mathrm{T}_6$ ($^3\mathrm{T}_{2g}$): 0.010 eV
$i = \nu_{47}$, $\alpha = \mathrm{T}_3$ ($^3\mathrm{T}_{1g}$, $\beta = \mathrm{T}_5$ ($^3\mathrm{T}_{2g}$): 0.013 eV
$i = \nu_{48}$, $\alpha = \mathrm{T}_1$ ($^3\mathrm{T}_{1g}$, $\beta = \mathrm{T}_4$ ($^3\mathrm{T}_{2g}$): 0.005 eV
$i = \nu_{48}$, $\alpha = \mathrm{T}_2$ ($^3\mathrm{T}_{1g}$, $\beta = \mathrm{T}_5$ ($^3\mathrm{T}_{2g}$): 0.004 eV
$i = \nu_{48}$, $\alpha = \mathrm{T}_2$ ($^3\mathrm{T}_{1g}$, $\beta = \mathrm{T}_6$ ($^3\mathrm{T}_{2g}$): 0.006 eV
$i = \nu_{48}$, $\alpha = \mathrm{T}_3$ ($^3\mathrm{T}_{1g}$, $\beta = \mathrm{T}_4$ ($^3\mathrm{T}_{2g}$): 0.010 eV
$i = \nu_{48}$, $\alpha = \mathrm{T}_3$ ($^3\mathrm{T}_{1g}$, $\beta = \mathrm{T}_5$ ($^3\mathrm{T}_{2g}$): 0.001 eV
$i = \nu_{48}$, $\alpha = \mathrm{T}_3$ ($^3\mathrm{T}_{1g}$, $\beta = \mathrm{T}_6$ ($^3\mathrm{T}_{2g}$): 0.003 eV
$i = \nu_{49}$, $\alpha = \mathrm{T}_1$ ($^3\mathrm{T}_{1g}$, $\beta = \mathrm{T}_5$ ($^3\mathrm{T}_{2g}$): 0.005 eV
$i = \nu_{49}$, $\alpha = \mathrm{T}_3$ ($^3\mathrm{T}_{1g}$, $\beta = \mathrm{T}_4$ ($^3\mathrm{T}_{2g}$): 0.002 eV
$i = \nu_{50}$, $\alpha = \mathrm{T}_2$ ($^3\mathrm{T}_{1g}$, $\beta = \mathrm{T}_4$ ($^3\mathrm{T}_{2g}$): 0.002 eV
$i = \nu_{50}$, $\alpha = \mathrm{T}_2$ ($^3\mathrm{T}_{1g}$, $\beta = \mathrm{T}_5$ ($^3\mathrm{T}_{2g}$): 0.003 eV
$i = \nu_{51}$, $\alpha = \mathrm{T}_3$ ($^3\mathrm{T}_{1g}$, $\beta = \mathrm{T}_6$ ($^3\mathrm{T}_{2g}$): 0.001 eV

First-order off-diagonal coefficients $\lambda_i^{(\alpha\beta)}$ for LVC-B3LYP* ($\geq$ 0.001 eV) quintet states:

$i = \nu_{5}$, $\alpha = \mathrm{Q}_1$ ($^5\mathrm{T}_{2g}$), $\beta = \mathrm{Q}_2$ ($^5\mathrm{T}_{2g}$): 0.011 eV
$i = \nu_{5}$, $\alpha = \mathrm{Q}_2$ ($^5\mathrm{T}_{2g}$), $\beta = \mathrm{Q}_3$ ($^5\mathrm{T}_{2g}$): 0.003 eV
$i = \nu_{6}$, $\alpha = \mathrm{Q}_1$ ($^5\mathrm{T}_{2g}$), $\beta = \mathrm{Q}_2$ ($^5\mathrm{T}_{2g}$): 0.013 eV
$i = \nu_{14}$, $\alpha = \mathrm{Q}_1$ ($^5\mathrm{T}_{2g}$), $\beta = \mathrm{Q}_3$ ($^5\mathrm{T}_{2g}$): 0.031 eV
$i = \nu_{23}$, $\alpha = \mathrm{Q}_2$ ($^5\mathrm{T}_{2g}$), $\beta = \mathrm{Q}_3$ ($^5\mathrm{T}_{2g}$): 0.003 eV
$i = \nu_{37}$, $\alpha = \mathrm{Q}_1$ ($^5\mathrm{T}_{2g}$), $\beta = \mathrm{Q}_2$ ($^5\mathrm{T}_{2g}$): 0.004 eV
$i = \nu_{40}$, $\alpha = \mathrm{Q}_1$ ($^5\mathrm{T}_{2g}$), $\beta = \mathrm{Q}_3$ ($^5\mathrm{T}_{2g}$): 0.027 eV
$i = \nu_{47}$, $\alpha = \mathrm{Q}_1$ ($^5\mathrm{T}_{2g}$), $\beta = \mathrm{Q}_3$ ($^5\mathrm{T}_{2g}$): 0.010 eV

Table S9: CASPT2 singlet-triplet SOC matrix elements (cm^{-1}) calculated at the FC geometry. For simplicity, only the FC adiabatic labels are used, for the corresponding diabatic labels see, e.g., Table S2. Elements not shown in the table are below 1 cm^{-1}.

SOC	$m_s = -1$		$m_s = 0$		$m_s = 1$	
	Re	Im	Re	Im	Re	Im
$\langle S_0 \vert \hat{H}_{SO} \vert T_1 \rangle$	−390.9				−390.9	
$\langle S_0 \vert \hat{H}_{SO} \vert T_2 \rangle$				552.9		
$\langle S_0 \vert \hat{H}_{SO} \vert T_3 \rangle$		−390.9				390.9
$\langle S_1 \vert \hat{H}_{SO} \vert T_2 \rangle$		−64.5				64.5
$\langle S_1 \vert \hat{H}_{SO} \vert T_3 \rangle$				−91.3		
$\langle S_1 \vert \hat{H}_{SO} \vert T_5 \rangle$		173.9				−173.9
$\langle S_1 \vert \hat{H}_{SO} \vert T_6 \rangle$				−245.9		
$\langle S_2 \vert \hat{H}_{SO} \vert T_1 \rangle$		−64.5				64.5
$\langle S_2 \vert \hat{H}_{SO} \vert T_3 \rangle$	64.5				64.5	
$\langle S_2 \vert \hat{H}_{SO} \vert T_4 \rangle$		−173.9				173.9
$\langle S_2 \vert \hat{H}_{SO} \vert T_6 \rangle$	−173.9				−173.9	
$\langle S_3 \vert \hat{H}_{SO} \vert T_1 \rangle$				−91.3		
$\langle S_3 \vert \hat{H}_{SO} \vert T_2 \rangle$	−64.5				−64.5	
$\langle S_3 \vert \hat{H}_{SO} \vert T_4 \rangle$				245.9		
$\langle S_3 \vert \hat{H}_{SO} \vert T_5 \rangle$	−173.9				−173.9	
$\langle S_3 \vert \hat{H}_{SO} \vert T_5 \rangle$	−173.9				−173.9	

Table S10: CASPT2 triplet-triplet SOC matrix elements (cm^{-1}) calculated at the FC geometry. For simplicity, only the FC adiabatic labels are used, for the corresponding diabatic labels see, e.g., Table S2. Elements not shown in the table are below 1 cm^{-1}.

SOC	$m_s = -1$		$m_s = 0$		$m_s = 1$	
	Re	Im	Re	Im	Re	Im
$\langle T_{1,-1}\|\hat{H}_{SO}\| T_2\rangle$				−100.8		
$\langle T_{1,-1}\|\hat{H}_{SO}\| T_3\rangle$		142.5				
$\langle T_{1,-1}\|\hat{H}_{SO}\| T_5\rangle$						
$\langle T_{1,-1}\|\hat{H}_{SO}\| T_6\rangle$		170.6				
$\langle T_{1,0}\|\hat{H}_{SO}\| T_2\rangle$		−100.8				−100.8
$\langle T_{1,0}\|\hat{H}_{SO}\| T_5\rangle$		120.6				120.6
$\langle T_{1,1}\|\hat{H}_{SO}\| T_2\rangle$				−100.8		
$\langle T_{1,1}\|\hat{H}_{SO}\| T_3\rangle$						−142.5
$\langle T_{1,1}\|\hat{H}_{SO}\| T_5\rangle$				120.6		
$\langle T_{1,1}\|\hat{H}_{SO}\| T_6\rangle$						−170.6
$\langle T_{2,-1}\|\hat{H}_{SO}\| T_3\rangle$			100.8			
$\langle T_{2,-1}\|\hat{H}_{SO}\| T_4\rangle$				120.6		
$\langle T_{2,-1}\|\hat{H}_{SO}\| T_6\rangle$			−120.6			
$\langle T_{2,0}\|\hat{H}_{SO}\| T_3\rangle$	−100.8				100.8	
$\langle T_{2,0}\|\hat{H}_{SO}\| T_4\rangle$		120.6				120.6
$\langle T_{2,0}\|\hat{H}_{SO}\| T_6\rangle$	120.6				−120.6	
$\langle T_{2,1}\|\hat{H}_{SO}\| T_3\rangle$			−100.8			
$\langle T_{2,1}\|\hat{H}_{SO}\| T_4\rangle$				120.6		
$\langle T_{2,1}\|\hat{H}_{SO}\| T_6\rangle$			120.6			
$\langle T_{3,-1}\|\hat{H}_{SO}\| T_4\rangle$		170.6				
$\langle T_{3,-1}\|\hat{H}_{SO}\| T_5\rangle$			−120.6			
$\langle T_{3,0}\|\hat{H}_{SO}\| T_5\rangle$	120.6				−120.6	
$\langle T_{3,1}\|\hat{H}_{SO}\| T_4\rangle$						−170.6
$\langle T_{3,1}\|\hat{H}_{SO}\| T_5\rangle$			120.6			
$\langle T_{4,-1}\|\hat{H}_{SO}\| T_5\rangle$				−98.2		
$\langle T_{4,-1}\|\hat{H}_{SO}\| T_6\rangle$		138.9				
$\langle T_{4,0}\|\hat{H}_{SO}\| T_5\rangle$		−98.2				−98.2
$\langle T_{4,1}\|\hat{H}_{SO}\| T_5\rangle$				−98.2		
$\langle T_{4,1}\|\hat{H}_{SO}\| T_6\rangle$						−138.5
$\langle T_{5,-1}\|\hat{H}_{SO}\| T_6\rangle$			98.2			
$\langle T_{5,0}\|\hat{H}_{SO}\| T_6\rangle$	−98.2				98.2	
$\langle T_{5,1}\|\hat{H}_{SO}\| T_6\rangle$			−98.2			

Table S11: CASPT2 triplet-quintet real SOC matrix elements (cm^{-1}) calculated at the FC geometry. For simplicity, only the FC adiabatic labels are used, for the corresponding diabatic labels see, e.g., Table S2. Elements not shown in the table are below 1 cm^{-1}.

Real SOC	$m_s = -2$	$m_s = -1$	$m_s = 0$	$m_s = 1$	$m_s = 2$
$\langle T_{2,-1}\|\hat{H}_{SO}\| Q_3\rangle$	335.4		136.9		
$\langle T_{2,0}\|\hat{H}_{SO}\| Q_3\rangle$		237.1		237.1	
$\langle T_{2,1}\|\hat{H}_{SO}\| Q_3\rangle$			136.9		335.4
$\langle T_{3,-1}\|\hat{H}_{SO}\| Q_2\rangle$	335.4		136.9		
$\langle T_{3,0}\|\hat{H}_{SO}\| Q_2\rangle$		237.1		237.1	
$\langle T_{3,1}\|\hat{H}_{SO}\| Q_2\rangle$			136.9		335.4
$\langle T_{5,-1}\|\hat{H}_{SO}\| Q_3\rangle$	201.5		82.3		
$\langle T_{5,0}\|\hat{H}_{SO}\| Q_3\rangle$		142.5		142.5	
$\langle T_{5,1}\|\hat{H}_{SO}\| Q_3\rangle$			82.3		201.5
$\langle T_{6,-1}\|\hat{H}_{SO}\| Q_2\rangle$	−201.5		−82.3		
$\langle T_{6,0}\|\hat{H}_{SO}\| Q_2\rangle$		−142.5		−142.5	
$\langle T_{6,1}\|\hat{H}_{SO}\| Q_2\rangle$			−82.3		−201.5

Table S12: CASPT2 triplet-quintet imaginary SOC matrix elements (cm^{-1}) calculated at the FC geometry. For simplicity, only the FC adiabatic labels are used, for the corresponding diabatic labels see, e.g., Table S2. Elements not shown in the table are below 1 cm^{-1}.

Imaginary SOC	$m_s = -2$	$m_s = -1$	$m_s = 0$	$m_s = 1$	$m_s = 2$
$\langle T_{1,-1}\|\hat{H}_{SO}\| Q_2\rangle$	335.4		−136.9		
$\langle T_{1,-1}\|\hat{H}_{SO}\| Q_3\rangle$		−335.4			
$\langle T_{1,0}\|\hat{H}_{SO}\| Q_2\rangle$		237.1		−237.1	
$\langle T_{1,0}\|\hat{H}_{SO}\| Q_3\rangle$			−387.3		
$\langle T_{1,1}\|\hat{H}_{SO}\| Q_2\rangle$			136.9		−335.4
$\langle T_{1,1}\|\hat{H}_{SO}\| Q_3\rangle$				−335.4	
$\langle T_{2,-1}\|\hat{H}_{SO}\| Q_1\rangle$	335.4		−136.9		
$\langle T_{2,0}\|\hat{H}_{SO}\| Q_1\rangle$		237.1		−237.1	
$\langle T_{2,1}\|\hat{H}_{SO}\| Q_1\rangle$			136.9		−335.4
$\langle T_{3,-1}\|\hat{H}_{SO}\| Q_1\rangle$		−335.4			
$\langle T_{3,0}\|\hat{H}_{SO}\| Q_1\rangle$			−387.3		
$\langle T_{3,1}\|\hat{H}_{SO}\| Q_1\rangle$				−335.4	
$\langle T_{4,-1}\|\hat{H}_{SO}\| Q_2\rangle$	201.5		−82.3		
$\langle T_{4,-1}\|\hat{H}_{SO}\| Q_3\rangle$		201.5			
$\langle T_{4,0}\|\hat{H}_{SO}\| Q_2\rangle$		142.5		−142.5	
$\langle T_{4,0}\|\hat{H}_{SO}\| Q_3\rangle$			232.7		
$\langle T_{4,1}\|\hat{H}_{SO}\| Q_2\rangle$			82.3		−201.5
$\langle T_{4,1}\|\hat{H}_{SO}\| Q_3\rangle$				201.5	
$\langle T_{5,-1}\|\hat{H}_{SO}\| Q_1\rangle$	−201.5		82.3		
$\langle T_{5,0}\|\hat{H}_{SO}\| Q_1\rangle$		−142.5		142.5	
$\langle T_{5,1}\|\hat{H}_{SO}\| Q_1\rangle$			−82.3		201.5
$\langle T_{6,-1}\|\hat{H}_{SO}\| Q_1\rangle$		−201.5			
$\langle T_{6,0}\|\hat{H}_{SO}\| Q_1\rangle$			−232.7		
$\langle T_{6,1}\|\hat{H}_{SO}\| Q_1\rangle$				−201.5	

Table S13: CASPT2 quintet-quintet real SOC matrix elements (cm^{-1}) calculated at the FC geometry. For simplicity, only the FC adiabatic labels are used, for the corresponding diabatic labels see, e.g., Table S2. Elements not shown in the table are below 1 cm^{-1}.

Real SOC	$m_s = -2$	$m_s = -1$	$m_s = 0$	$m_s = 1$	$m_s = 2$
$\langle Q_{2,-2}\|\hat{H}_{SO}\| Q_3\rangle$		−84.6			
$\langle Q_{2,-1}\|\hat{H}_{SO}\| Q_3\rangle$	84.6		−103.6		
$\langle Q_{2,0}\|\hat{H}_{SO}\| Q_3\rangle$		103.6		−103.6	
$\langle Q_{2,1}\|\hat{H}_{SO}\| Q_3\rangle$			103.6		−84.6
$\langle Q_{2,2}\|\hat{H}_{SO}\| Q_3\rangle$				84.6	

Table S14: CASPT2 quintet-quintet imaginary SOC matrix elements (cm^{-1}) calculated at the FC geometry. For simplicity, only the FC adiabatic labels are used, for the corresponding diabatic labels see, e.g., Table S2. Elements not shown in the table are below 1 cm^{-1}.

Imaginary SOC	$m_s = -2$	$m_s = -1$	$m_s = 0$	$m_s = 1$	$m_s = 2$
$\langle Q_{1,-2}\|\hat{H}_{SO}\| Q_2\rangle$		84.6			
$\langle Q_{1,-2}\|\hat{H}_{SO}\| Q_3\rangle$	−169.2				
$\langle Q_{1,-1}\|\hat{H}_{SO}\| Q_2\rangle$	84.6		103.6		
$\langle Q_{1,-1}\|\hat{H}_{SO}\| Q_3\rangle$		−84.6			
$\langle Q_{1,0}\|\hat{H}_{SO}\| Q_2\rangle$		103.6		103.6	
$\langle Q_{1,1}\|\hat{H}_{SO}\| Q_2\rangle$			103.6		84.6
$\langle Q_{1,1}\|\hat{H}_{SO}\| Q_3\rangle$				84.6	
$\langle Q_{1,2}\|\hat{H}_{SO}\| Q_2\rangle$				84.6	
$\langle Q_{1,2}\|\hat{H}_{SO}\| Q_3\rangle$					169.2

S3 Diabatic and Adiabatic/Spin-Diabatic Potentials

We here analyze the diabatic potentials and coupling strengths for the three different models LVC-B3LYP*, VCHAM-B3LYP*, and VCHAM-CASPT2, which the dynamics simulations are based on. We first consider the antisymmetric Fe-N stretching modes. Figure S1 shows the diabatic PESs along the component of the degenerate antisymmetric Fe-N stretching vibrations (ν_{13} or ν_{14}), which accounts for stronger vibronic effects in the diabatic potentials, quantified by the linear on-diagonal $\kappa_i^{(\alpha)}$ constants. As seen in Figure S1, the singlet and triplet PESs along ν_{13}/ν_{14} (light blue and green) split, and importantly, the $^1T_{1g}$ and $^3T_{2g}$ potentials cross in regions that are easily accessible by the wavepacket in the initially excited $^1T_{1g}$ states. This is the reason for the fast (∼100−300 fs) singlet-triplet ISC. The overall agreement for the PESs shown in Figure S1 is rather good, which supports the adequacy of B3LYP*, as well as the LVC approach for constructing diabatic states. In order to analyze the singlet-triplet ISC, it is also important to consider the magnitude of nonadiabatic couplings between the singlet excited states; this is also shown in Figure S1 for the two antisymmetric Fe-N stretching modes, ν_{13} and ν_{14}. As seen in the figure, the magnitude of singlet couplings is comparable for the three methods, with the weakest coupling strengths for LVC-B3LYP*. However, even in the case of these weaker couplings ($0.01-0.05$ eV), the LVC-B3LYP* $^1T_{1g}/^3T_{2g}$ crossings are easily accessible, as reflected by the ∼200−300 fs singlet-triplet ISC timescales obtained by the corresponding TSH and QD simulations (see Figures 4a,c in the article).

In Figure S2, we present the PESs along the totally symmetric Fe-N stretching mode ν_{15}, which, as discussed in Section 4.1 in the article, is the key mode for the quintet states (double occupation of e_g^* orbitals). Similarly to the case of the antisymmetric Fe-N stretching modes, the overall agreement is rather good. There are two aspects, though, for which the DFT/TD-DFT (B3LYP*) and CASPT2 results differ: i) the separation of the two triplet manifolds $^3T_{1g}$ and $^3T_{2g}$ is roughly twice as large for CASPT2 (Figure S2, panel c) than for B3LYP* (Figure S2, panels a and b), and ii) in contrast to CASPT2, for B3LYP*, the perfect triple degeneracy of the quintet $^5T_{2g}$ states along ν_{15} is not maintained, which is a consequence of the description of the lowest quintet by as single Slater determinant (unrestricted DFT) and the other two by quintet TD-DFT. In addition, for LVC-B3LYP* (Figure S2, panel a), the HS-LS energy gap along ν_{15} is lowered by ∼0.2−0.3 eV. However, as is clear from Figure 4 in the article, these variations in the PESs along ν_{15} do not lead to any qualitative differences in the simulated dynamics. We note that nonadiabatic couplings are negligible for mode ν_{15}.[2]

Figures S3–S5 and S6–S8 show the B3LYP* (DFT/TD-DFT) adiabatic/spin-diabatic PESs of $[Fe(NCH)_6]^{2+}$, obtained by the VCHAM and LVC methods, respectively. In the case of VCHAM, both quantum chemistry data points and energy curves obtained by the diagonalization of the diabatic Hamiltonian (fits) are shown. We note again that for VCHAM, modes ν_{13}, ν_{14} (antisymmetric modes), we had to include three additional singlet excited states (i.e., the three components of the $^1T_{2g}$ manifold), as the $^1T_{1g}$-$^1T_{2g}$ states interact along ν_{13} and ν_{14}; this was not necessary for LVC, as the quantum chemistry calculations were only performed in the vicinity of the FC geometry, where no

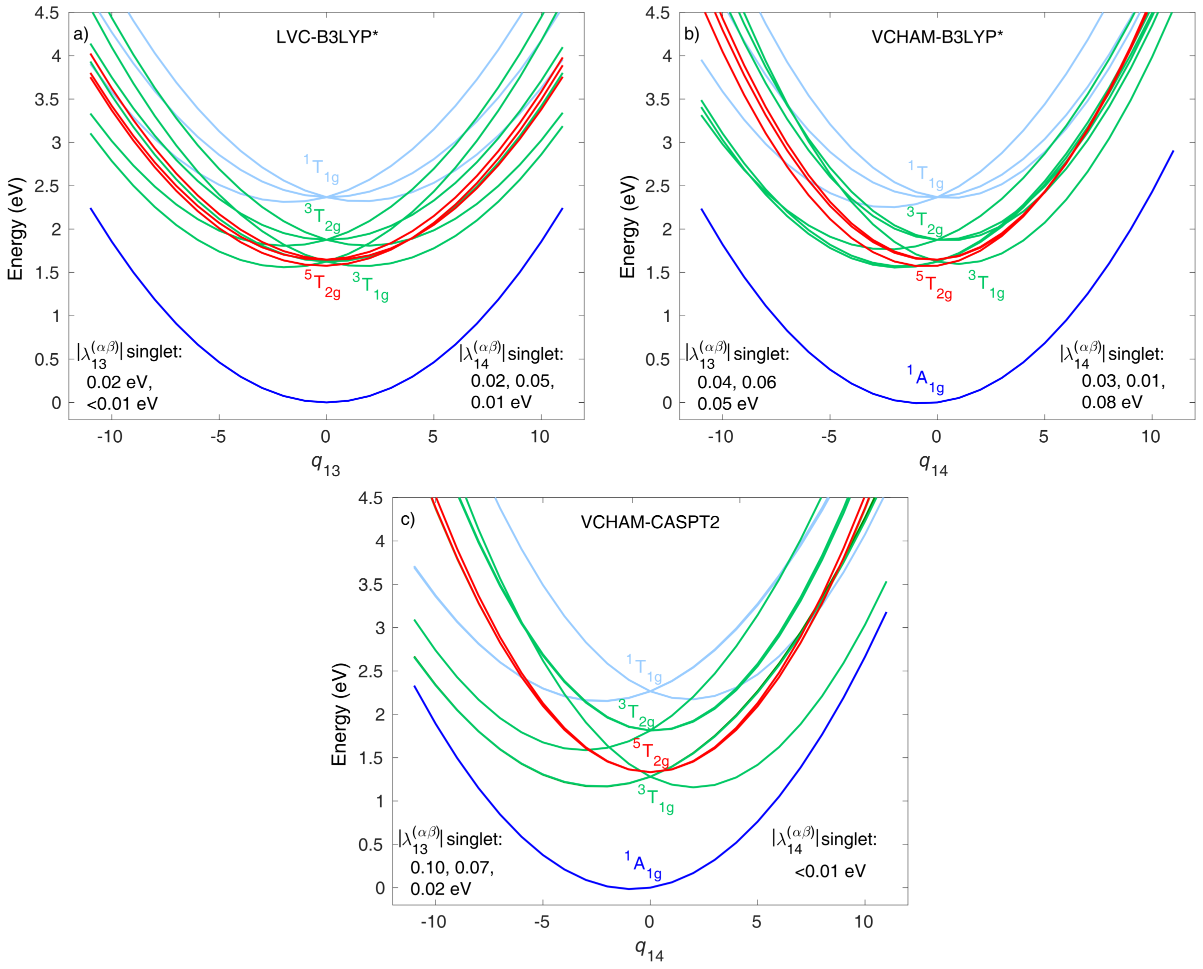

Figure S1: Diabatic 1D PESs of $[Fe(NCH)_6]^{2+}$ along the antisymmetric Fe-N stretching mode (ν_{13} or ν_{14}) that is dominant based on the values of the linear diagonal constants $\kappa_i^{(\alpha)}$ values. The magnitude of the singlet linear off-diagonal $\lambda_i^{(\alpha\beta)}$ values for modes ν_{13} and ν_{14} are also shown. The three panels show the PESs for a) LVC-B3LYP*, b) VCHAM-B3LYP*, and c) VCHAM-CASPT2.

$^1T_{1g}$-$^1T_{2g}$ interaction occurs.

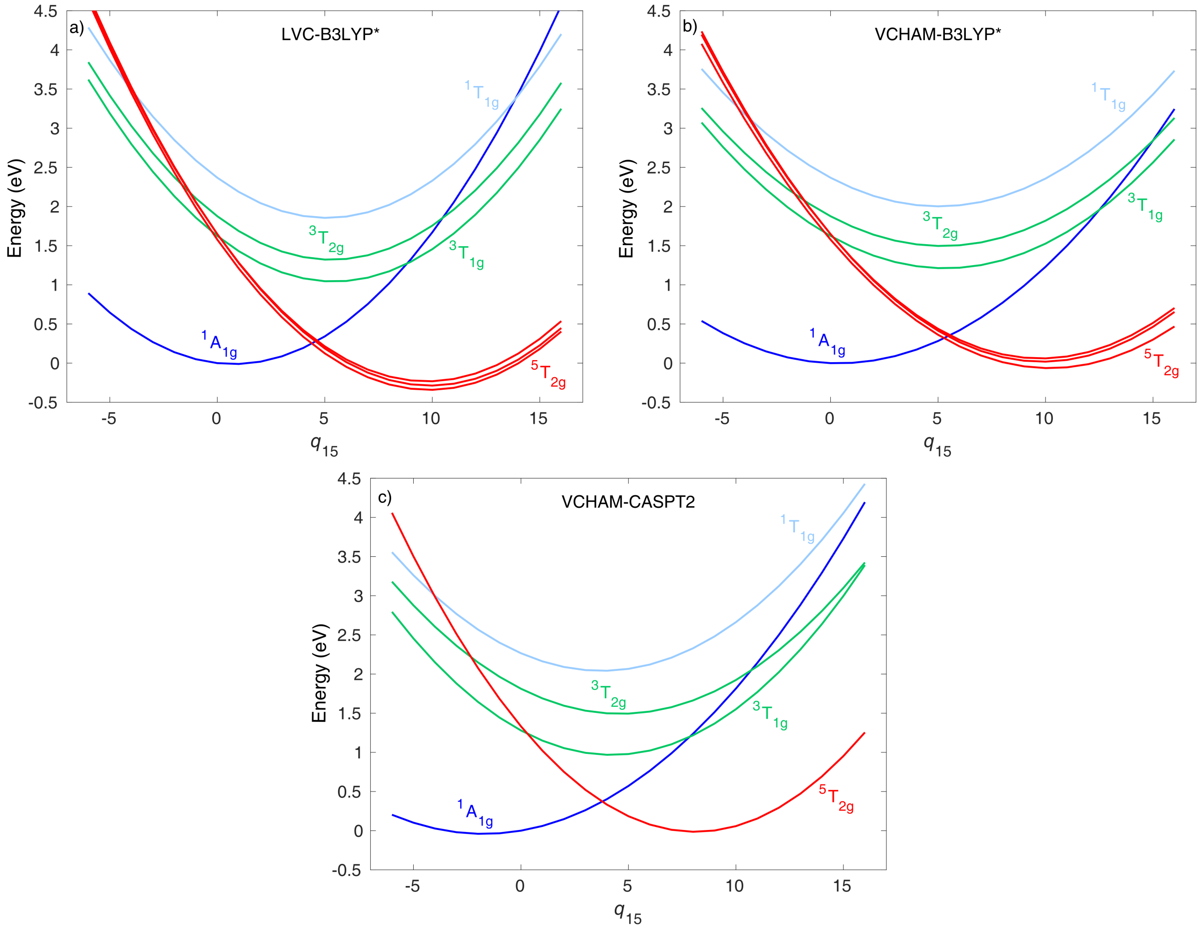

Figure S2: Diabatic 1D PESs of $[Fe(NCH)_6]^{2+}$ along the totally symmetric Fe-N stretching mode ν_{15}. The three panels show the PESs for a) LVC-B3LYP*, b) VCHAM-B3LYP*, and c) VCHAM-CASPT2.

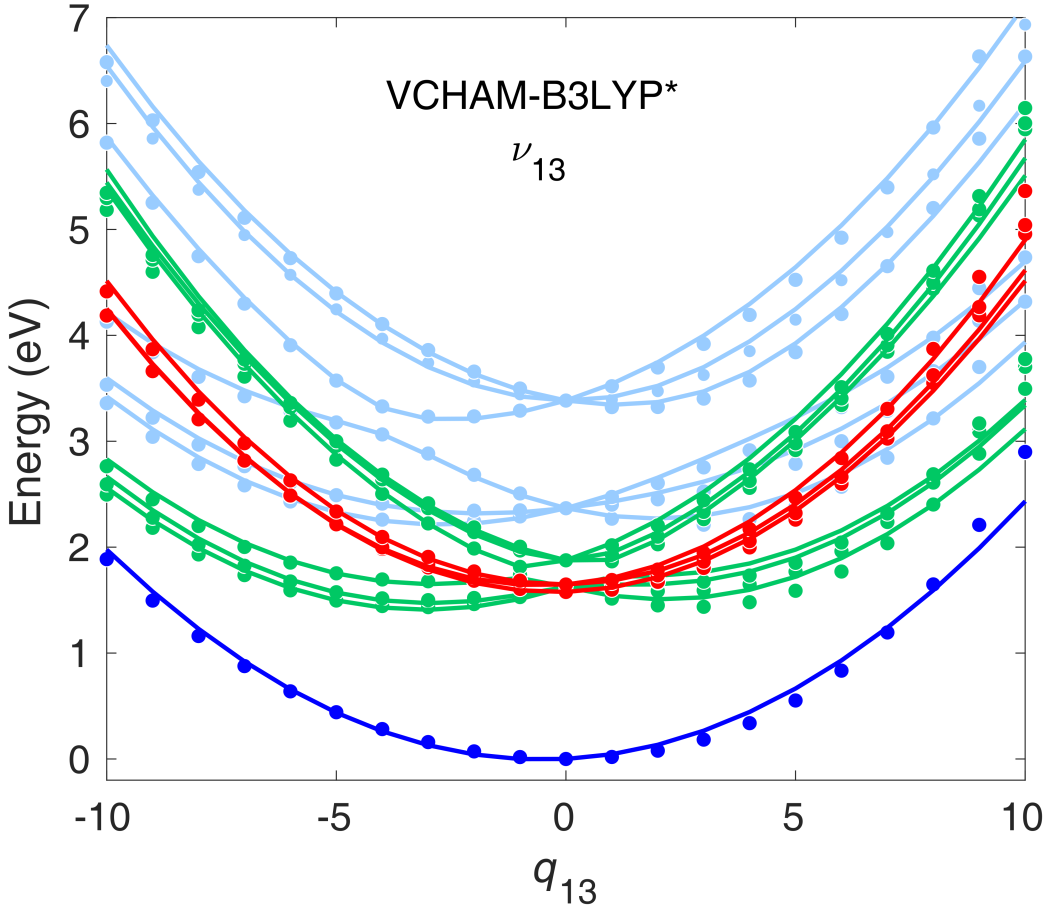

Figure S3: DFT/TD-DFT (B3LYP*) adiabatic/spin-diabatic potential energy surfaces of $[Fe(NCH)_6]^{2+}$ along ν_{13}. Obtained by using the VCHAM method. The dots are the energies calculated by quantum chemistry, while the lines correspond to their VCHAM fit. The following colour code is used: blue – ground state (S_0), light blue – singlet excited states (S_1–S_6; $^1T_{1g}$ and $^1T_{2g}$), green – triplet excited states T_1–T_6; $^3T_{1g}$ and $^3T_{2g}$), red – quintet excited states (Q_1–Q_3; $^5T_{2g}$).

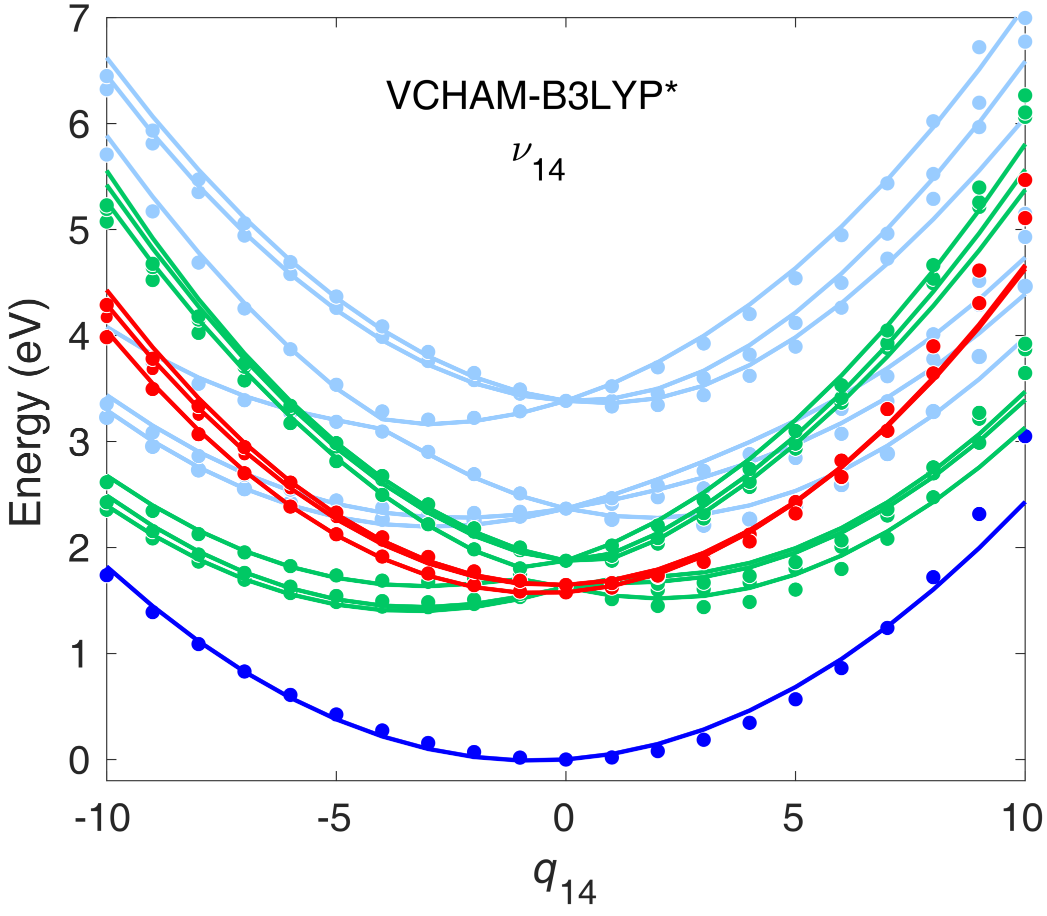

Figure S4: DFT/TD-DFT (B3LYP*) adiabatic/spin-diabatic potential energy surfaces of $[Fe(NCH)_6]^{2+}$ along ν_{14}. Obtained by using the VCHAM method. The dots are the energies calculated by quantum chemistry, while the lines correspond to their VCHAM fit. The following colour code is used: blue – ground state (S_0), light blue – singlet excited states (S_1–S_6; $^1T_{1g}$ and $^1T_{2g}$), green – triplet excited states T_1–T_6; $^3T_{1g}$ and $^3T_{2g}$), red – quintet excited states (Q_1–Q_3; $^5T_{2g}$).

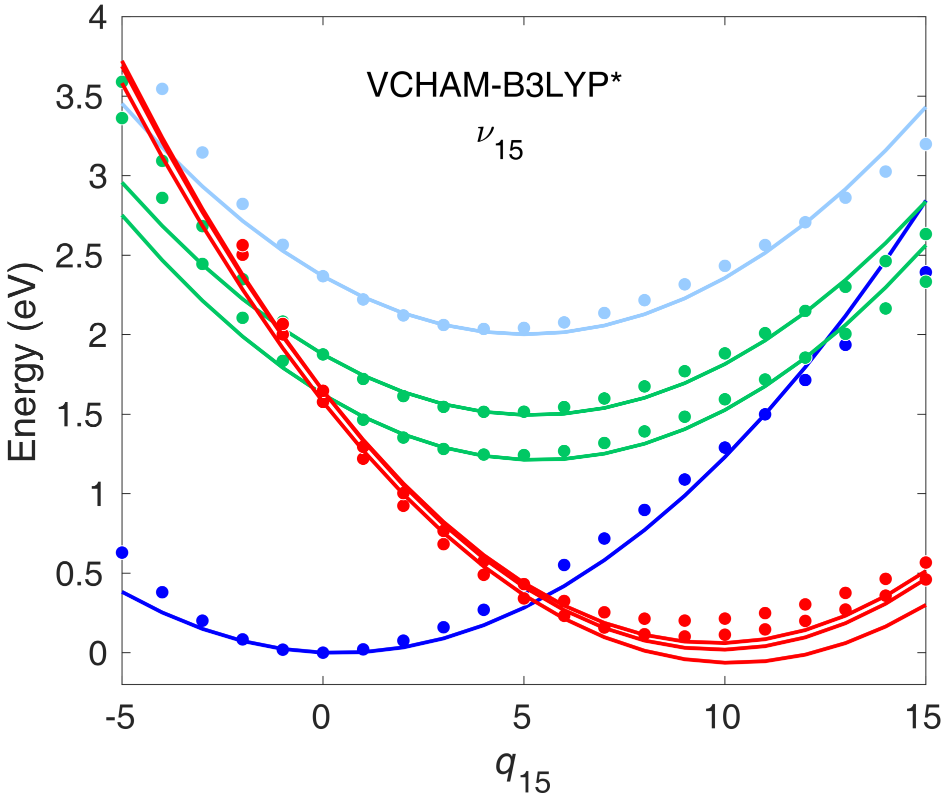

Figure S5: DFT/TD-DFT (B3LYP*) adiabatic/spin-diabatic potential energy surfaces of $[Fe(NCH)_6]^{2+}$ along ν_{15}. Obtained by using the VCHAM method. The dots are the energies calculated by quantum chemistry, while the lines correspond to their VCHAM fit. The following colour code is used: blue – ground state (S_0), light blue – singlet excited states (S_1–S_6; $^1T_{1g}$ and $^1T_{2g}$), green – triplet excited states T_1–T_6; $^3T_{1g}$ and $^3T_{2g}$), red – quintet excited states (Q_1–Q_3; $^5T_{2g}$).

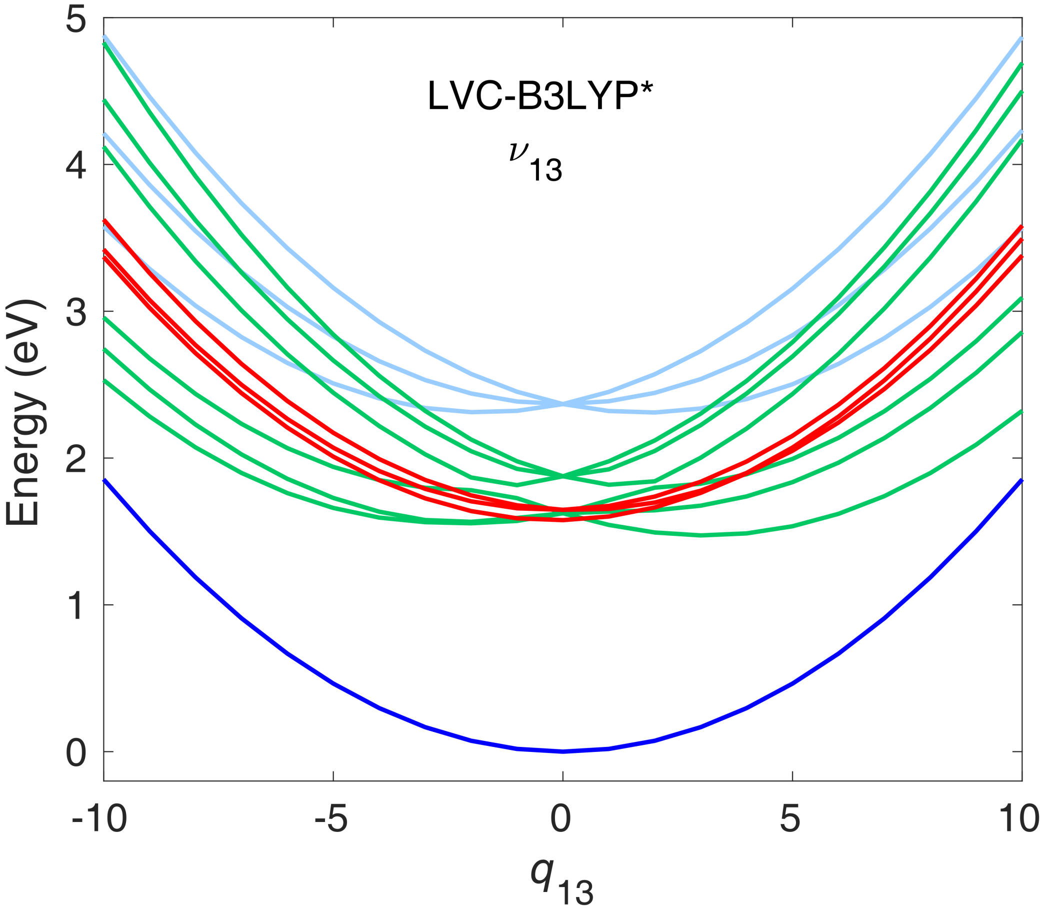

Figure S6: DFT/TD-DFT (B3LYP*) adiabatic/spin-diabatic potential energy surfaces of $[Fe(NCH)_6]^{2+}$ along ν_{13}. Obtained by using the LVC method. The following colour code is used: blue – ground state (S_0), light blue – singlet excited states (S_1–S_6; $^1T_{1g}$ and $^1T_{2g}$), green – triplet excited states T_1–T_6; $^3T_{1g}$ and $^3T_{2g}$), red – quintet excited states (Q_1–Q_3; $^5T_{2g}$).

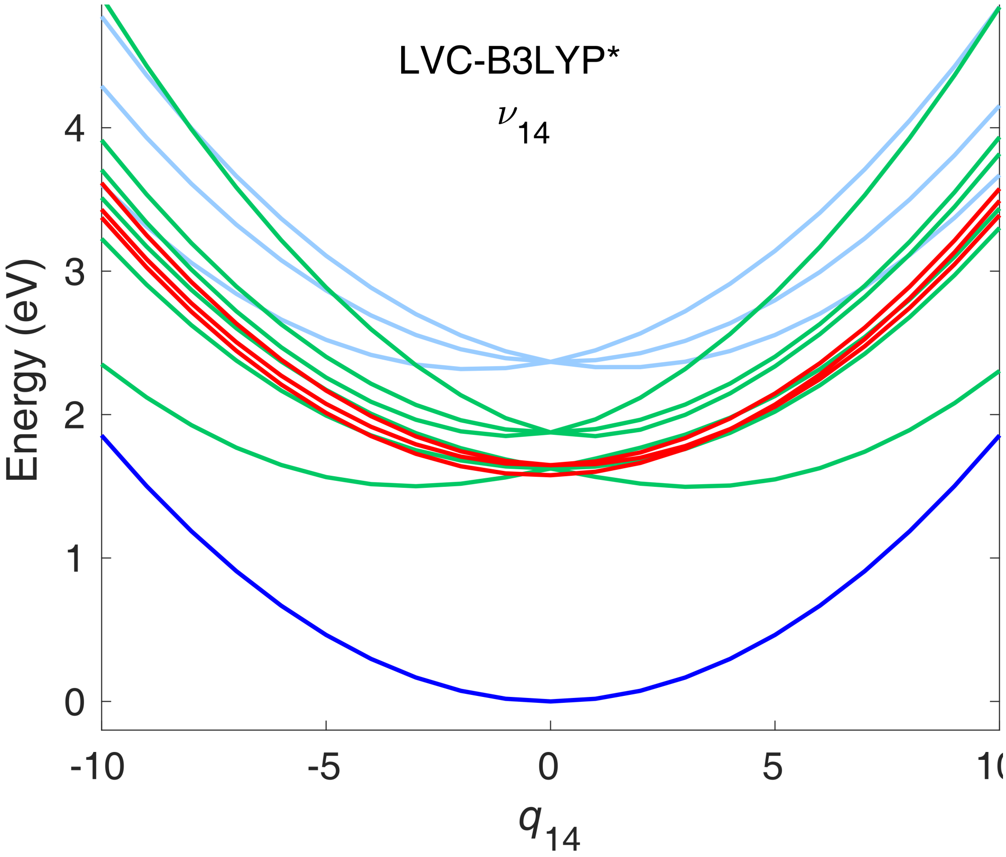

Figure S7: DFT/TD-DFT (B3LYP*) adiabatic/spin-diabatic potential energy surfaces of $[Fe(NCH)_6]^{2+}$ along ν_{14}. Obtained by using the LVC method. The following colour code is used: blue – ground state (S_0), light blue – singlet excited states (S_1–S_6; $^1T_{1g}$ and $^1T_{2g}$), green – triplet excited states T_1–T_6; $^3T_{1g}$ and $^3T_{2g}$), red – quintet excited states (Q_1–Q_3; $^5T_{2g}$).

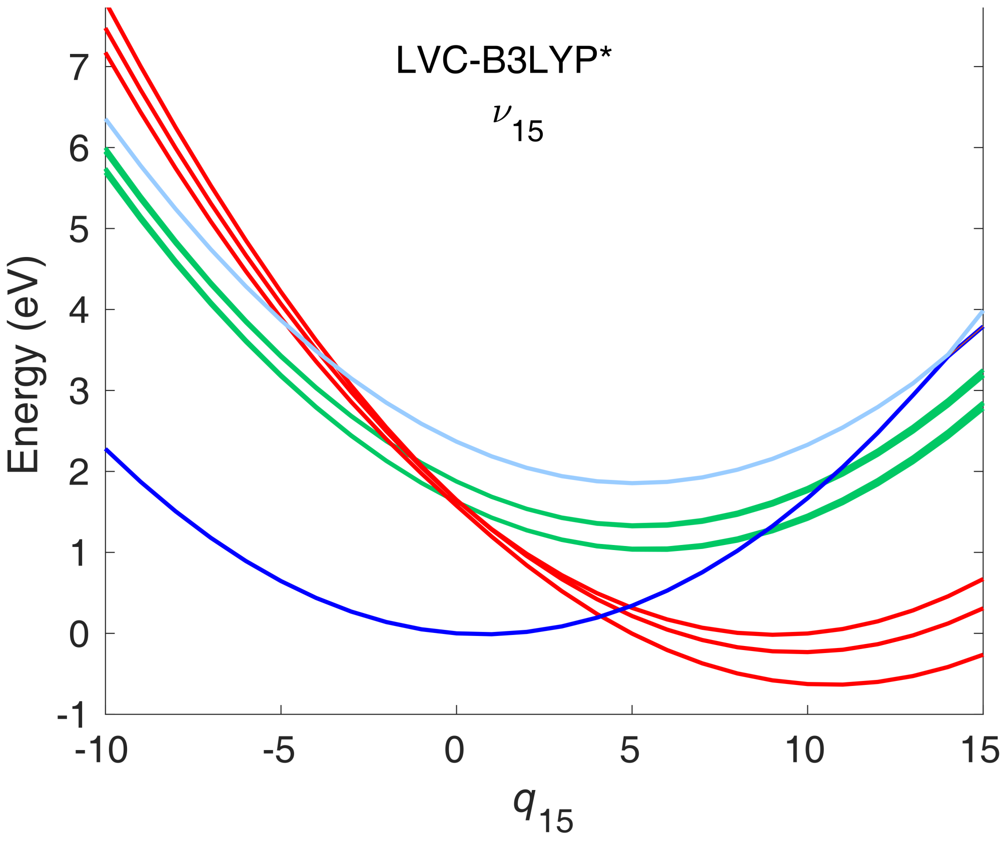

Figure S8: DFT/TD-DFT (B3LYP*) adiabatic/spin-diabatic potential energy surfaces of $[Fe(NCH)_6]^{2+}$ along ν_{15}. Obtained by using the LVC method. The following colour code is used: blue – ground state (S_0), light blue – singlet excited states (S_1–S_6; $^1T_{1g}$ and $^1T_{2g}$), green – triplet excited states T_1–T_6; $^3T_{1g}$ and $^3T_{2g}$), red – quintet excited states (Q_1–Q_3; $^5T_{2g}$).

S4 Comparison of B3LYP* and CASPT2 QD

Figure S9 shows the 3D QD populations dynamics obtained by the VCHAM-B3LYP* and VCHAM-CASPT2[1] models. As is clear from the figure, the triplet IC is affected by the utilized quantum chemistry method. This is explained by the nearly twice as large $^3T_{1g}$-$^3T_{2g}$ energy gap (see Table S2), which is consistent with the differences observed in Figure S9.

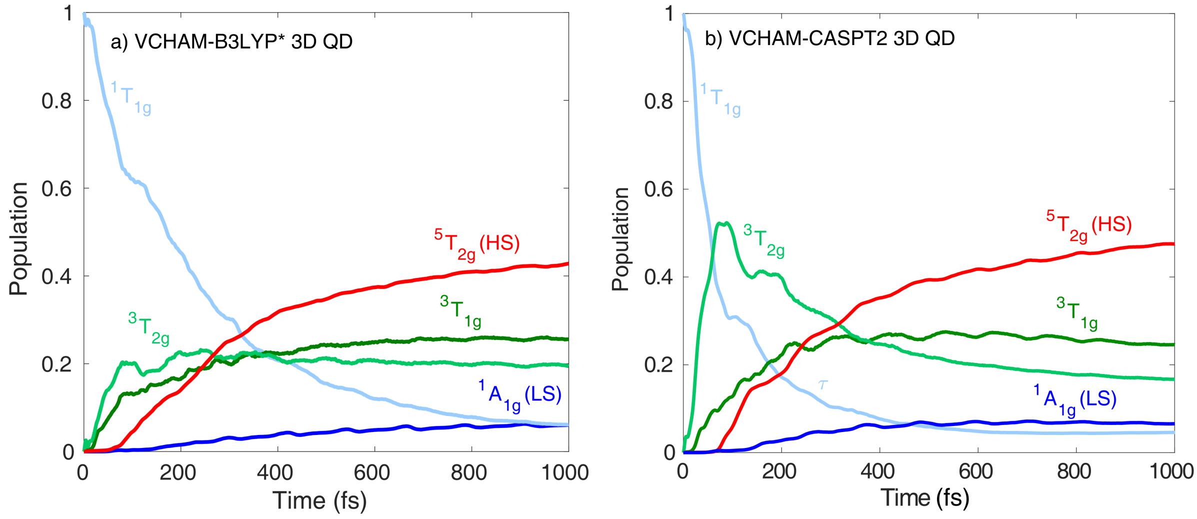

Figure S9: 3D QD diabatic population dynamics obtained by a) VCHAM-B3LYP* and VCHAM-CASPT2.[1]

.

S5 Description of Vibrational Dynamics by TSH and QD

We now assess how the TSH and QD methods describe the nuclear dynamics of photoexcited $[Fe(NCH)_6]^{2+}$. For this analysis, we utilize our LVC-B3LYP* Hamiltonian, which allows direct comparison of the TSH and QD results. Figure S10 shows the TSH nuclear dynamics along ν_{13} and ν_{15}. As seen in Figure S10, panel a, and also the single trajectory in Figure 5a in the article, $^1A_{1g} \rightarrow {}^1T_{1g}$ excitation immediately activates ν_{13}. Propagation occurs equally towards positive and negative q_{13} values, which is due to the antisymmetric character of ν_{13}, which breaks the threefold symmetry of $^1T_{1g}$. This has the consequence that the average displacement cancels out (two components with the same magnitude but opposite sign), and thus $\langle q_{13} \rangle$ is zero (thick black line in Figure

S10a). This is in line with a bifurcating wavepacket created by nonadiabatic coupling between the $^1T_{1g}$ components. The ensemble of trajectories dephases and broadens in $\sim 100 - 200$ fs, which is in agreement with our QD simulations on CASPT2 PESs.[1] From ca. 400 fs, weak, slowly growing oscillations appear in the average $\langle q_{13} \rangle$, which we attribute to the growing quintet population; this is supported by the asymmetry in the distribution of triplet-quintet hopping geometries projected to ν_{13} shown in Figure 7a in the article.

The trajectories along the breathing mode ν_{15} are displayed in Figure S10, panel b, which are very different from those along ν_{13}, discussed above. The dynamics along ν_{15} are characterized by coherent oscillations (breathing vibrations) with a period of ca. 100 fs with rather slow coherence decay. Initially, the oscillations turn at ca. $q_{15} = -2$ and $q_{15} = 12$, which, according to the PESs along ν_{15} (Figure S8) are consistent with vibrations along ν_{15} in the singlet ($^1T_{1g}$) and triplet ($^3T_{1g}$, $^3T_{2g}$) excited states, respectively. At the second oscillation period, a new component appears with an outer turning point at ca. $q_{15} = 20$, which is assigned to the quintet ($^5T_{2g}$) states. At the next inner turning point, a third component arises between ca. $q_{15} = -7$ and $q_{15} = -8$, which we assign to the ground state $^1A_{1g}$. These ν_{15} structural features are clearly characteristic of the electronic character (shown in Figure S10, panel b), reflecting the e_g^* occupation ($^1T_{1g}$, $^3T_{1g}$, $^3T_{2g}$ – single, $^5T_{2g}$ - double, $^1A_{1g}$ - zero occupation). The coherently oscillating average $\langle q_{15} \rangle$ slowly shifts towards higher q_{15} values, which, as seen from Figure S10, panel b, is due to the $^5T_{2g}$ component, whose relative weight (i.e., population) gradually increases in line with rising quintet population. We note that oscillations with the same period appear in the corresponding quintet population curve (Figure 4a in the article, red), evidencing that the triplet-quintet transition is controlled by breathing mode ν_{15}.

Figure S11 shows the QD vibrational dynamics, characterized by the wavepacket centroids $\langle q_i \rangle$ and widths dq_i, along ν_{13} and ν_{15}. Here, $\langle q_i \rangle$ and dq_i were calculated by a weighted sum over the electronic states, with the weights being the relative populations. As in the case of TSH (Figure S10), the QD vibrational dynamics are dominated by the coherently oscillating breathing motion ν_{15}, with the same period. The shift towards higher $\langle q_{15} \rangle$ values due to the population of quintet states is again observed, and loss of coherence is even slower than for the TSH results shown in Figure S10, panel b, in fact, it is negligible. This is reasonable because our TSH model is full dimensional, which thus allows interaction of all modes, while our QD Hamiltonian includes only the three Fe-N stretching modes. Interestingly, our VCHAM-CASPT2 QD simulation with these three modes leads to a significantly faster coherence decay.[3] This means that the in the present case, the influence of vibronic effects, which are stronger for CASPT2, on the coherence decay is stronger than the presence of the bath of vibrational modes. Finally, the wavepacket along the antisymmetric mode ν_{13} broadens similarly and on the same timescale as in the case of TSH dynamics. On the other hand, the centroid $\langle q_{13} \rangle$ does not cancel initially but exhibits small oscillations that are slowly damped, in contrast to the slowly growing oscillations for TSH in Figure S10, panel a. The reason for this difference is that the preparation of the initial state is different: while a single *diabatic* $^1T_{1g}$ component for QD, an *adiabatic*, i.e., a mixture of $^1T_{1g}$ states for TSH. Note,

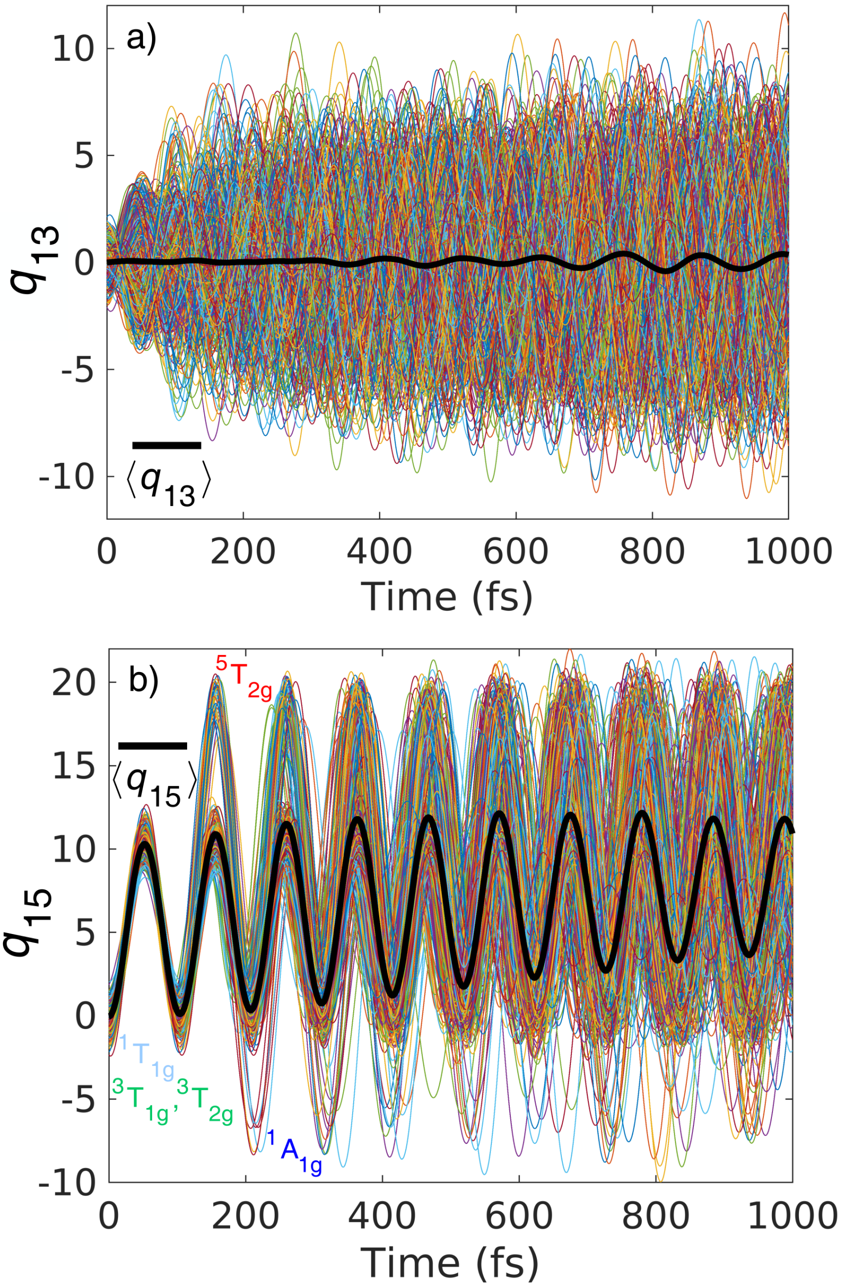

Figure S10: Nuclear dynamics along a) ν_{13} and b) ν_{15}, as obtained from the LVC-B3LYP* TSH simulations. All 500 trajectories are shown as well as their average (thick black line). The trajectories along ν_{15} are assigned to different electronic states; this assignment is given in panel b).

however, that due to the degeneracy of $^1T_{1g}$ components within octahedral symmetry, this difference in the excitation scheme should not show up in any physical observable; indeed, the corresponding TSH and QD population dynamics show very good agreement (Figures 4a and 4c in the article).

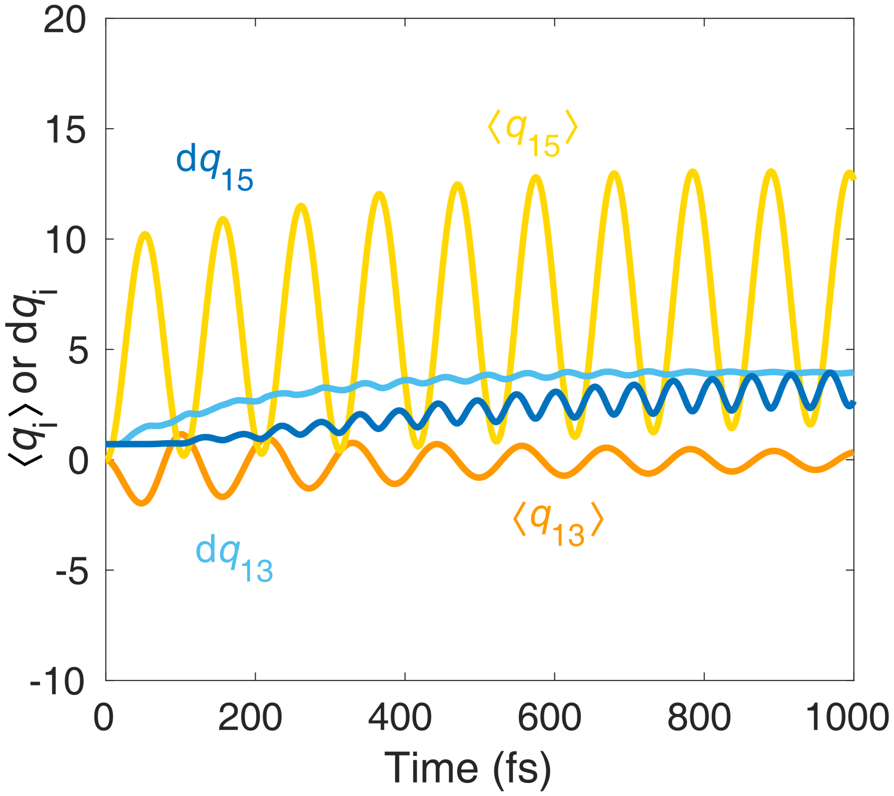

Figure S11: Nuclear dynamics along ν_{13} and ν_{15}, as obtained from the LVC-B3LYP* QD simulations. $\langle q_i \rangle$ and dq_i denote the centroid and width of the wavepacket, respectively.

References

[1] Pápai, M. *Inorg. Chem.* **2021**, *60*, 13950–13954.

[2] All off-diagonal $\lambda_i^{(\alpha\beta)}$ values are all negligible for mode ν_{15} with a single exception: ∼0.03 eV, for quintet states obtained by LVC-B3LYP*. This exceptional case likely occurs due to the quintet unrestricted DFT/TD-DFT treatment.

[3] In ref. 1, the coherence decay is analyzed for the $^5T_{2g}$ states, but the same trends hold for the full wavepacket.
